# Regulation of Ion Homeostasis for Enhanced Tumor Radio‐Immunotherapy

**DOI:** 10.1002/advs.202304092

**Published:** 2023-09-22

**Authors:** Rui Qian, Xuan Yi, Teng Liu, Hua Chen, Yuhong Wang, Lin Hu, Lingchuan Guo, Kai Yang, Haijun Deng

**Affiliations:** ^1^ Department of General Surgery and Guangdong Provincial Key Laboratory of Precision Medicine for Gastrointestinal Tumor, Nanfang Hospital, The First School of Clinical Medicine Southern Medical University Guangzhou 510000 China; ^2^ School of Pharmacy, Jiangsu Key Laboratory of Inflammation and Molecular Drug Targets Nantong University Nantong Jiangsu 226001 China; ^3^ State Key Laboratory of Radiation Medicine and Protection, School of Radiation Medicine and Protection and School for Radiological and Interdisciplinary Sciences (RAD‐X), Collaborative Innovation Center of Radiation Medicine of Jiangsu Higher Education Institutions Soochow University Suzhou Jiangsu 215123 China; ^4^ Department of Pathology, The First Affiliated Hospital of Soochow University Soochow University Suzhou 215005 China

**Keywords:** calcium ion overload, immunogenic cell death, radio‐immunotherapy, tumor acidity neutralization

## Abstract

Intra/extracellular ion content affects the growth and metastasis of tumor cells, as well as the efficacy of various antitumor therapies. Herein, a carbonic anhydrase inhibitor (CAI) is loaded onto pH‐responsive calcium carbonate (CaCO_3_) nanoparticles and then modify theses nanoparticles with liposomes to obtain biocompatible CaCO_3_/CAI@Lipsome (CCL) for enhance tumor radio‐immunotherapy. CCL can specially decompose in tumor microenvironment, releasing calcium ion (Ca^2+^) and CAI, as well as increasing the pH value of extracellular fluid. CAI restrains the flow of hydrogen ion (H^+^) inside and outside the tumor cells, resulting in the reversal of tumor acidic microenvironment and the increase of intracellular H^+^, both of which can improve the sensitivity of tumor to radiotherapy. Afterward, the increased intracellular H^+^ together with radiotherapy‐causes reactive oxygen species promotes calcium influx, leading to cellular calcium overload. Moreover, the CCL‐tailored content of H^+^ and Ca^2+^ strengthens radiotherapy‐induced immunogenic cell death and dendritic cell maturation, amplifying systemic anti‐tumor adaptive immunity. Meanwhile, macrophages in the CCL‐treated tumors are polarized from pro‐tumor M2 to anti‐tumor M1 under X‐ray exposure, owing to the neutralization of tumor acidic microenvironment and enhances Ca^2+^ content. Therefore, multi‐directional regulation of the intra/extra tumor cell pH/calcium by simple nano‐preparation would provide a powerful way to improve the efficacy of radio‐immunotherapy.

## Graphical abstract

Carbonic anhydrase inhibitor‐loaded and pH‐responsive calcium carbonate (CaCO_3_) nanoparticles are designed to regulate the pH and calcium ions in tumor cells intra/extracellularly. The nanoparticles improves the efficacy of radiotherapy and strengthen the radiotherapy‐induced abscopal effect, resulting in perfect inhibition of primary and distant tumors.

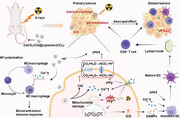

## 1 Introduction

Various ions inside and outside tumor cells, including hydrogen ion (H^+^), calcium ion (Ca^2+^), cupric ion (Cu^2+^), iron ion (Fe^3+^), manganese ion (Mn^2+^) and aluminium ion (Al^3+^), play an important role in the tumorigenesis, development and treatment sensitivity/resistance.^[^ ^1^ ^]^ Thereinto, H^+^ is best known for the specifically higher concentration in tumors than normal tissues, which is ascribed to the preference of tumor cells for glycolysis than oxidative phosphorylation and the subsequent accumulation of lactic acid.^[^ ^2^ ^]^ However, the level of H^+^ inside and outside tumor cells is not consistently high. The extracellular moiety of carbonic anhydrase (CA) specifically catalyzes the hydration of carbon dioxide to generate bicarbonate ions and hydrogen ions, wherein bicarbonate ions would flow into cells with the help of Na^+^/HCO_3_^−^ co‐transporter while hydrogen ions are left outside, resulting in the consumption of intracellular hydrogen ions and the accumulation of extracellular hydrogen ions.^[^ ^3^ ^]^ The elevated extracellular hydrogen ions contribute to the acidic tumor microenvironment, which prevents the infiltration of anti‐tumor immune cells, promotes the migration and invasion of tumor cells, and leads to resistance to various treatments.^[^ ^4^ ^]^ On the contrary, it has been discovered that the increase of H^+^ content inside tumor cells would accelerate the influx of calcium ions, making tumors more sensitive to multiple treatments.^[^ ^5^ ^]^

Recently, the function of Ca^2+^ in tumor therapy has received wide attention due to its special role in calcium death and anti‐tumor immune regulation.^[^ ^6^ ^]^ Normally, exogenous calcium supplementation is often ineffective to kill tumor cells, because excess calcium is always discharged from the intercellular space via various calcium channels.^[^ ^7^ ^]^ However, reactive oxygen species (ROS) in cells triggered by some drugs or treatments may disrupt calcium homeostasis and cause intracellular calcium overload, which amplifies the production of ROS and finally leads to mitochondrial damage and dysfunction of cell metabolism^.[^ ^8^ ^]^ Moreover, Ca^2+^ is able to upregulate the expression of various damage‐associated molecular patterns (DAMPs), including calreticulin (CRT), heat shock protein 70 (HSP70), and heat shock protein 90 (HSP90), in the dying tumor cells,^[^ ^9^ ^]^ further facilitating dendritic cell (DC) maturation and tumor antigen presentation.^[^ ^10^ ^]^ In addition, Ca^2+^ is involved in the macrophage polarization from pro‐tumor M2 to anti‐tumor M1, improving antitumor immunotherapy by macrophages.^[^ ^11^ ^]^ In this way, calcium supplementation, in combination with ROS‐generating anti‐tumor treatments, may not only induce “calcium death” of tumor cells, but also strengthen the systemic anti‐tumor innate and adaptive immune response.^[^ ^12^ ^]^ Since radiotherapy could produce abundant ROS in tumors while feebly induce antitumor immune response,^[^ ^13^ ^]^ the regulation of H^+^ and Ca^2+^ homeostasis in tumor cells would provide benefits for radio‐immunotherapy.^[^ ^14^ ^]^

**Scheme 1:**
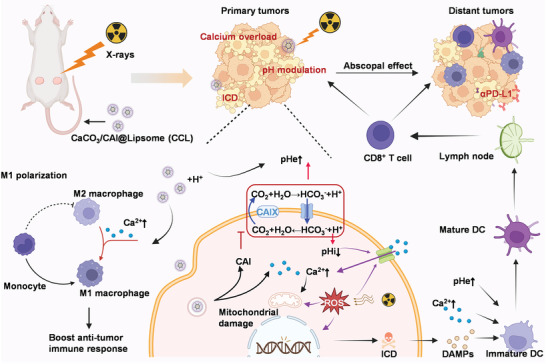
Schematic illustration of CaCO_3_/CAI@Liposome (CCL) reprograming the H^+^ and Ca^2+^ content inside and outside tumor cells for enhanced radio‐immunotherapy. CCL could degrade in the acidic tumor microenvironment, resulting in the depletion of H^+^ and the release of Ca^2+^/CAI. The released CAI specifically inhibited CA on tumor cell membrane, reversing tumor acidic microenvironment and aggravating intracellular metabolic acidosis. Meanwhile, the enhanced intracellular H^+^ and X‐rays exposure further induced calcium overload. Specially, CCL‐mediated radiotherapy caused significant immunogenic cell death (ICD) to promote DC maturation and then reinforced T cell‐mediated antitumor immunotherapy. The macrophages were also polarized from M2 to M1 phenotype with the help of plentiful Ca^2+^ in the mice treated with CCL‐mediated radiotherapy.

In this study, we synthesized liposome‐coated carbonic anhydrase inhibitor (CAI)‐loaded calcium carbonate nanoparticles (CaCO_3_/CAI@Liposome (CCL)) as pH‐responsive nanomedicine to reprogram the H^+^ and Ca^2+^ content inside and outside tumor cells for enhanced radio‐immunotherapy (**Scheme** **1**). CAI loading could be easily realized by adding CAI into the Ca^2+^ solution during the gaseous diffusion process of naked calcium carbonate synthesis. The obtained nanoparticles were then modified with liposomes to increase their biocompatibility, yielding CaCO_3_/CAI@Liposome (CCL). CCL could degrade in the acidic solution, resulting in the depletion of H^+^ and the release of Ca^2+^ and CAI. The released CAI specifically inhibited CA on tumor cell membrane, reversing tumor acidic microenvironment and aggravating intracellular metabolic acidosis. Meanwhile, the enhanced intracellular H^+^ and X‐rays exposure further induced calcium overload. Therefore, this strategy based on CCL‐disrupted ion homeostasis in tumor cells improved the sensitivity of tumor tissue to radiotherapy, as well as the immune‐activation capacity of radiotherapy. Specially, CCL‐mediated radiotherapy caused significant immunogenic cell death (ICD) to promote DC maturation, further reinforcing T cell‐mediated antitumor immunotherapy. Meanwhile, the macrophages were also polarized from M2 to M1 phenotype in the mice treated with CCL‐mediated radiotherapy. The tumor growth was significantly inhibited and the survival time was prolonged after CCL‐mediated radiotherapy, especially together with the intravenous injection of αPD‐L1. Therefore, we developed a practical and effective strategy to boost the therapeutic effect of tumor radio‐immunotherapy by regulating the ion content inside and outside tumor cells.

## 2 Results and Discussion

### 2.1 Synthesis and Characterization of CaCO_3_/CAI@Liposome

**Figure 1:**
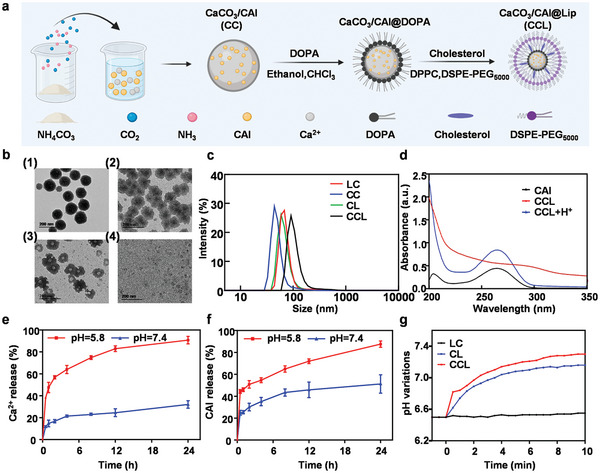
Synthesis and characterization of CaCO_3_/CAI@Liposome (CCL). a) Schematic diagram showing the synthesis and modification of CCL nanoparticles by a one‐pot method. b) Transmission electron microscopy images of 1) CaCO_3_/CAI (CC), 2) CCL (pH = 7.4), 3) CCL (pH = 6.5) and 4) CCL (pH = 5.8). The scale bar was 200 nm. c) Hydrodynamic size distribution of Lip/CAI (LC), CaCO_3_@Liposome (CL), CC, and CCL. d) UV–vis absorption spectra of free CAI, CCL, CCL+H^+^. e,f) The release curve of Ca^2+^ (e) and CAI (f) from CCL in solutions with different pH values. Data are presented as mean ± s.d. (n = 3) g) In vitro acidity neutralization profiles of CL, LC, and CCL.

The calcium carbonate nanoparticles loaded with CAI acetazolamide (CaCO_3_/CAI, CC) were synthesized using a one‐pot method.^[^ ^15^ ^]^ To obtain better biocompatibility, the yielded CC was then modified with 1, 2‐dioleoyl‐sn‐glycero‐3‐phosphate (sodium salt) (DOPA) and DSPE‐PEG_5000_ to form liposome structure on the surface (CaCO_3_/CAI@Liposome, CCL) via thin‐film dispersion‐membrane extrusion technique (**Figure** **1**a). Transmission electron microscopy (TEM) images revealed that CC nanoparticles were uniform spheres with an average size of ≈100 nm (Figure 1b). After modification with liposomes, a thin layer with optical contrast was visualized on the surface of CC, indicating the establishment of lipid bilayer. Notably, in the process of liposome coating, ≈27.4% ± 0.9% of calcium carbonate nanoparticles were lost owing to the multiple extrusion procedures through the membrane. Interestingly, the obtained CCL nanoparticles exhibited partial degradation at pH 6.5 followed by the formation of cavity structures, while most of the CCL was found to be completely degraded at pH 5.8, suggesting the pH‐responsive degradation property of CCL nanoparticles (Figure 1b). According to DLS measurement, we found that CCL (115.5 ± 14.0 nm) showed slightly bigger hydrodynamic size than that of CAI‐loaded liposome (CL) (96.6 ± 4.4 nm) and liposome‐coated CaCO_3_ (LC) (86.4 ± 10.7 nm), while being much bigger than CC (81.7 ± 11.8 nm) (Figure 1c). The sizes of these nanoparticles were suitable for enhanced permeability and retention (EPR) effect of tumors. Although CCL did not show a characteristic peak of CAI (265 nm) in a neutral environment, the addition of H^+^ could lead to the appearance of this peak in the UV–vis‐NIR spectrum, suggesting the successful loading of CAI by CCL (Figure 1d). The loaded CAI was within the CaCO_3_ nanoparticles at pH 7.4, which blocked the UV absorption of CAI, while free CAI could leak out from the CCL after acid treatment, reflecting the pH‐responsive release of CAI from CCL. By calculation, the CAI loading efficiency and encapsulation efficiency of CaCO_3_/CAI@lip nanoparticles were 7.93% ± 0.18% and 61.7% ± 1.1%, respectively. Specifically, we incubated CCL at different pH values including pH 5.8 and pH 7.4, and collected the released Ca^2+^ and CAI by dialysis to analyze pH‐dependent Ca^2+^/CAI release. As shown in Figure 1e, 90.8±3.8% of Ca^2+^ would be gradually released from CCL at pH 5.8 in 24 h, while only 32.0±3.9% of Ca^2+^ was detected by inductively coupled plasma optical emission spectrometry (ICP‐OES) at pH 7.4. Similarly, 87.6±3.3% of CAI was released after 24 h incubation at pH 5.8, while only 51.2±9.5% of CAI was released at pH 7.4 (Figure 1f). Meanwhile, LC and CCL could rapidly increase the pH value of solution after the reaction with H^+^, indicating that our designed CaCO_3_ based nanoparticles owned excellent proton neutralization ability (Figure 1 g).

### 2.2 The Regulation of pH and Ca^2+^ Content by CCL

**Figure 2:**
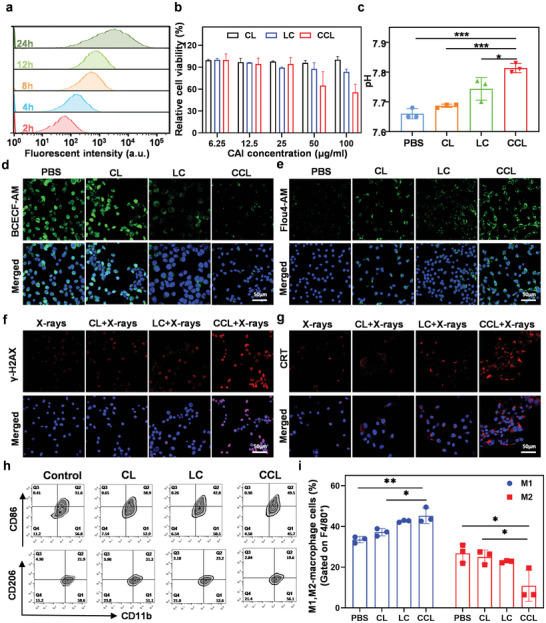
The regulation of pH value and Ca^2+^ content by CCL. a) Time‐dependent cellular uptake of DiD‐labeled CCL by CT26 cells. b) The viability of CT26 cells incubated with different concentrations of CL, LC, or CCL. Data are presented as mean ± s.d. (n = 3). c) The pH values of culture medium of CT26 cells incubated with PBS, CL, LC, or CCL. The dose of the calcium and CAI was 400 and 100 µg ml^−1^. Data are presented as mean ± s.d. (n = 3). d,e) BCECF‐AM (d) and Flou4‐AM (e) fluorescent images of CT26 cells incubated with PBS, CL, LC, or CCL. f,g) γ‐H2AX (f) and CRT (g) fluorescent images of CT26 cells incubated with PBS, CL, LC, or CCL under X‐rays exposure. The scale bar was 50 µm. The dose of the calcium and CAI was 400 and 100 µg ml^−1^. h,i) Representative flow cytometry plots (h) and statistical analysis (i) of macrophage polarization levels of RAW 264.7 treated with PBS, CL, LC, or CCL. Data are presented as mean ± s.d. (n = 3). All data are analyzed by one‐way ANOVA. *, P<0.05, **, P < 0.01, ***, P < 0.001, ****, P < 0.0001.

First, we explored the cellular uptake of 1, 1′‐dioctadecyl‐3,3,3′,3′‐tetramethylindodicarbocyanine,4‐chlorobenzenesulfonate salt (DiD)‐labeled CCL by murine CT26 cells. As shown in **Figure** **2**a and Figure S1 (Supporting Information), CCL could be gradually taken up by CT26 cells, showing the perfect drug‐delivery capacity of CCL. Next, the viability of NIH‐3T3 or CT26 cells was respectively measured after the co‐incubation with CL, LC, or CCL for 24 h. These three nanoparticles showed scarcely any toxicity to NIH‐3T3 cells, suggesting the high bio‐safety of our obtained nanoparticles (Figure S2, Supporting Information). On the contrary, it was found that CCL significantly induced CT26 cells death compared with CL or LC alone, indicating that the simultaneous presence of Ca^2+^ and CAI could significantly inhibit the viability of cancer cells (Figure 2b). Meanwhile, CCL significantly inhibited the clonogenic ability of CT26 cells compared with the other control groups (Figure S3, Supporting Information). In order to explain this phenomenon, we tested the pH of supernatant cell medium after co‐incubation with CL, LC, or CCL for 24 h. CaCO_3_ nanoparticles with weak alkalinity could increase the pH of cell medium by the reaction with extracellular H^+^, indicating that CCL might be used to reverse the tumor acid microenvironment (Figure 2c). Moreover, a pH fluorescent probe of 2′,7′‐bis‐(2‐carboxyethyl)−5‐(and‐6)‐carboxyfluorescein, acetoxymethyl ester (BCECF‐AM), which was able to be cleaved by intracellular esterase to form green fluorescence‐emitting BCECF, was incubated with CT26 cells for 24 h. The fluorescence intensity of BCECF, which was positively correlated with the intracellular pH, was observed by confocal fluorescence microscopy. Both LC and CCL could decrease the intracellular pH by inhibiting the expression of carbonic anhydrase (Figure S4, Supporting Information). Due to the degradation of CL in lysosomes, CL had no effect on intracellular pH (Figure 2d; Figure S5a, Supporting Information). In addition, the content of intracellular Ca^2+^ was also tested by Ca^2+^ fluorescent probe Fluo‐4 AM, which could be cleaved by intracellular esterase and bound to Ca^2+^ to form green fluorescence‐emitting Fluo‐4. All of CL, LC, and CCL could increase the intracellular Ca^2+^ concentration, while CCL induced the strongest elevation of Ca^2+^ concentration as compared to CL and LC (Figure 2e; Figure S5b, Supporting Information). Therefore, it could be inferred that CaCO_3_ enhanced the intra‐ and extracellular Ca^2+^ content and CAI caused the low intracellular pH to induce Ca^2+^ influx, resulting in the increased intracellular Ca^2+^ concentration in CCL‐treated cells. Taken together, after endocytosis of CCL by CT26 cells, the contained CaCO_3_ nanoparticles could decompose in the acidic tumor microenvironment and release a large amount of Ca^2+^ to increase the intracellular Ca^2+^ concentration. Meanwhile, the loaded CAI was also released and inhibited the carbonic anhydrase, leading to a decrease in intracellular pH, which might further induce the influx of Ca^2+^ and result in calcium overload. In short, CCL reversed the acidic tumor microenvironment and increased the content of H^+^ and Ca^2+^ in the tumor cells. In this way, the changed intra‐ and extracellular ions content could further enhance the ROS level in the CCL‐treated CT26 cells (Figure S6, Supporting Information).

### 2.3 Radio‐Sensitization Effect of CCL In Vitro

According to the published literatures, a decrease in extracellular H^+^ content would partly reverse radiation resistance, while an increase in intracellular H^+^ and Ca^2+^ content could improve the radio‐sensitivity.^[^ ^16^ ^]^ Motivated by the capability of CCL to tailor the content of H^+^ and Ca^2+^ inside and outside tumor cells, we further investigated the enhancement of radiotherapy effects by CCL in vitro. Immunofluorescent imaging of γ‐H2AX in cells demonstrated that CL, LC, or CCL themselves as well as X‐rays irradiation alone induced negligible DNA damage in CT26 cells (Figure 2f; Figure S7a, Supporting Information). Meanwhile, CT26 treated with combined nanomaterials and X‐rays irradiation demonstrated notable fluorescence of γ‐H2AX, showing the highest fluorescence intensity in CCL plus X‐rays irradiation (Figure 2f; Figure S7b, Supporting Information). Additionally, the clone formation experiments demonstrated that all of CL, LC, and CCL enhanced the suppress effect of X‐rays on the clone formation of CT26 cells (Figure S8, Supporting Information). Among them, CCL in combination with X‐rays irradiation inhibited cancer proliferation most potently, which could be ascribed to the excess ROS caused by X‐rays as well as the intracellular H^+^ elevation‐mediated enhancement of calcium influx by CCL.

In addition to the direct killing effect of cancer cells, radiotherapy has been well recognized for the complicated influence on tumor immune microenvironment.^[^ ^17^ ^]^ Therefore, we then tested whether these nanomaterials would confer benefits for radiotherapy‐triggered systemic antitumor immune response. First, the expression of calreticulin (CRT) on cell surface was characterized by confocal imaging to test the extent of ICD after each treatment (Figure 2g; Figure S9, Supporting Information). The highest fluorescence intensity of CRT was achieved by the combined treatment of CCL and X‐ray irradiation, suggesting that this treatment is promising for facilitating the recognition of tumor specific antigens by DC cells and subsequently the elimination of cancer cells by cytotoxic T cells (CTLs). Next, considering the reported influence of radiotherapy on the polarization of macrophages,^[^ ^18^ ^]^ we wondered if these nanomaterials with ability of tailoring ion content inside and outside tumor cells had any effect on macrophage polarization. The macrophage phenotypes after different treatments were analyzed by flow cytometry, showing that all of LC, CL, and CCL enhanced the proportion of M1 macrophages and decreased the proportion of M2 macrophages, among which CCL achieved the highest M1/M2 ratio (Figure 2h,i).

Taken together, CCL enhanced the radio‐sensitivity of tumor cells via reducing the extracellular H^+^ and increasing the intracellular content of H^+^ and Ca^2+^. Subsequently, the intracellular Ca^2+^ overload could also facilitate the death of tumor cells. Relying on this ion regulation strategy, CCL could promote the antigen presentation and inflammatory macrophage polarization to facilitate anti‐tumor immunotherapy.

### 2.4 The In Vivo Behavior and Tumor Microenvironment Regulation of CCL

**Figure 3:**
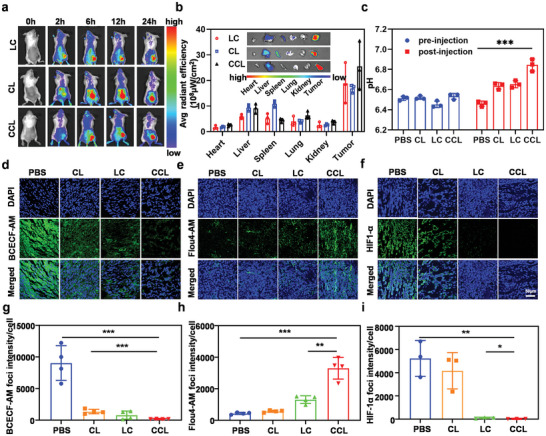
The in vivo behavior and tumor microenvironment regulation of CCL. a) IVIS fluorescent images of CT26‐bearing mice at different time points after i.v. injection of DiD‐labeled CL, LC, or CCL. The dose of the calcium and CAI was 10 and 2.5 mg kg^−1^. b) The ex vivo fluorescent images (insert) and semi‐quantitative analysis of main organs and tumors collected at 24 h after i.v. injection of DiD‐labeled CL, LC, or CCL. Data are presented as mean ± s.d. (n = 3). c) The pH values of tumors on mice before and after injection of PBS, CL, LC, or CCL. Data are presented as mean ± s.d. (n = 3). d–f) BCECF‐AM (d), Flou4‐AM (e), and HIF‐1α (f) fluorescent images of the tumors collected from mice with indicated treatment. The scale bar was 50 µm. The dose of the calcium and CAI was 10 and 2.5 mg kg^−1^. g–i) Corresponding semi‐quantitative analysis of BCECF‐AM (g), Flou4‐AM (h), and HIF‐1α (i) fluorescence intensity in tumors collected from mice with indicated treatment. Data are presented as mean ± s.d. (n = 4). All data are analyzed by one‐way ANOVA. *, P<0.05, **, P < 0.01, ***, P < 0.001, ****, P < 0.0001.

The tumor accumulation of CCL was studied in CT26 tumor‐bearing mice. First, the fluorescein DiD was inserted into the lipid bilayer of liposomes to obtain DiD‐LC, DiD‐CL, and DiD‐CCL. Then the fluorescent imaging system was used to observe the bio‐distribution of DiD in tumor‐bearing mice after the intravenous injection of these nanoparticles. These nanoparticles quickly accumulated into the tumors within 2 h after intravenous injection and retained in the tumor sites for at least 24 h (**Figure** **3**a). Afterwards, the major organs and the tumors were collected and imaged at 24 h post injection. It was demonstrated that the fluorescence intensity in the tumor site was higher than that in other normal organs, showing perfect tumor‐targeting capacity of these nanoparticles and thus providing a solid foundation for effective tumor theranostics (Figure 3b). Moreover, we collected the major organs and tumors at 2 and 24 h post nanoparticles injection and measured the DiD content after homogenizing the tissues. Similar to the fluorescent images, the fluorescent intensity in homogenized tissues suggested that these nanoparticles quickly entered the tumor sites and showed high tumor accumulation at 2 and 24 h post injection (Figure S10a,b, Supporting Information). In contrast, the fluorescence intensity in the liver and spleen homogenates was relatively higher than that in the fluorescent images, which is reasonable due to the fluorescence quenching of DiD in the liver and spleen of mice. Additionally, the Ca^2+^ content in major organs and tumors 24 h post injection of PBS or CCL was determined by ICP‐OES. CCL injection could significantly enhance the Ca^2+^ content in the tumor tissue, which was conducive to the tumor therapy (Figure S11, Supporting Information). Finally, in order to ensure the in vivo bio‐safety of CCL nanoparticles, we collected the blood samples from healthy mice treated with PBS or CCL (The dose of the calcium and CAI was 10 mg kg^−1^ and 2.5 mg kg^−1^.) at 3 days post injection and performed the blood routine and blood biochemistry analysis. The tested blood indicators showed no obvious side effect of CCL injection, probably owing to the biodegradability of CCL (Figure S12, Supporting Information) and the high LD50 of CAI (over 3.0 g kg^−1^, the data was obtained from “Drug Future Chemical Toxicity Database” (https://www.drugfuture.com/toxic/)).

Since CCL could regulate the content of H^+^ and Ca^2+^ inside and outside tumor cells, we then measured the pH value of these tumors with different treatments including CL, LC, and CCL. To explore the effect of various nanoparticles on the pH value of tumor microenvironment, twelve mice with bilateral tumors were randomly divided into four groups (n = 3). One side of the tumor was excised and broken down into a cellular homogenate with scissors, and its pH value was measured with a pH meter. After the wound was stitched, these mice were also intravenously injected with PBS, LC, CL or CCL, and the pH value of the contralateral tumor was measured by the same method described above at 24 h post injection. As shown in Figure 3c, all of CL, LC, and CCL were able to increase the pH value of the tumor microenvironment, while CCL was the most effective. Meanwhile, we examined the intracellular H^+^ and Ca^2+^ content of frozen slices of tumors using pH fluorescent probe BCECF AM and Ca^2+^ fluorescent probe Fluo‐4 AM. The results showed an increase in intracellular Ca^2+^ concentration and a decrease in intracellular pH in CCL‐treated mice, which was consistent with cellular experiments, indicating that CCL could induce the same changes in H^+^ and Ca^2+^ content in vivo as in vitro (Figure 3d–h). Additionally, hypoxia‐inducible factor 1α (HIF‐1α) plays an important role in the adaptation process of tumor cells and is overexpressed in acidic microenvironments or anaerobic conditions. The up‐regulation of this protein predicts a poor prognosis of cancer treatment and promotes tumor recurrence and metastasis .^[^ ^19^ ^]^ Therefore, we also studied the expression of HIF‐1α at tumor sites after different treatments. It was found that HIF‐1α immunofluorescence sections of tumor tissue confirmed that CL, LC, and CCL could reduce the expression of HIF‐1α by regulating intracellular and extracellular pH, while CCL was the most efficient (Figure 3f,i).

### 2.5 The Tumor Growth Inhibition by CCL

**Figure 4:**
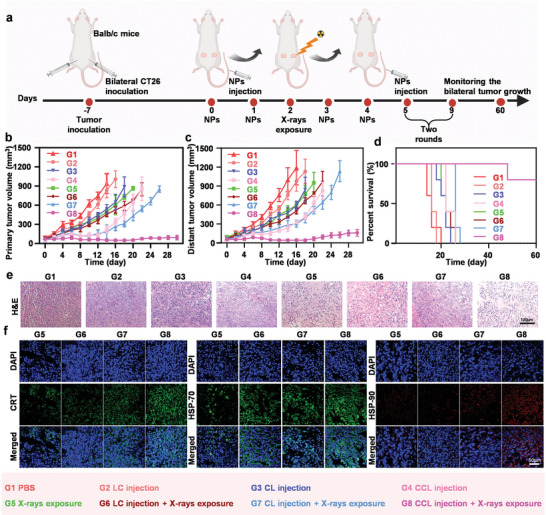
The tumor growth inhibition by CCL. a) Schematic diagram of the experimental schedule. The dose of the calcium and CAI was 10 and 2.5 mg kg^−1^ for each single injection. b) Tumor volume of primary tumors on mice in various groups. c) Tumor volume of distant tumors on mice in various groups. d) Survival curves of mice after various treatments. Data are presented as mean ± s.d. (n = 5). e) H&E staining of primary tumors on mice with indicated treatment. The scale bar was 100 µm. f) Immunofluorescent slices of the CRT, HSP70 and HSP90 expression in primary tumors. The scale bar was 50 µm.

Next, CCL was used for tumor radio‐sensitization, and the antitumor immune‐stimulatory effects by CCL‐mediated radiotherapy was also explored in vivo. We conducted an antitumor therapy study using Balb/c mice with bilateral CT26 tumor as animal models. When tumor volume reached ≈75 mm^3^, forty Balb/c mice with bilateral tumor were randomly divided into 8 groups (n = 5 per group) and received the following treatments: G1) PBS; G2) LC injection; G3) CL injection; G4) CCL injection; G5) X‐rays exposure; G6) LC injection + X‐rays exposure; G7) CL injection + X‐rays exposure; G8) CCL injection + X‐rays exposure (**Figure** **4**a). CL, LC, and CCL were intravenously injected at day 0, 1, 3, 4, 5, 6, 8, 9, and the dose of the calcium and CAI was 10 and 2.5 mg kg^−1^, respectively. X‐rays exposure was conducted at day 2 and 7, and the dose of the X‐rays was 6 Gy. According to the record of primary tumor volumes, it was found that plain LC and CL had little effect on tumor growth, while individual CCL and X‐rays exposure realized a moderate antitumor effect. All these three nanoparticles could enhance the therapeutic effect of radiotherapy, and the tumors were almost completely suppressed when CCL and X‐rays exposure were used in combination (Figure 4b; Figure S13a, Supporting Information). Similarly, the trend of tumor growth inhibition was the same between the distant tumor and primary tumor in different groups. Tumor volume was significantly reduced only when tumor‐bearing mice were injected with CCL and exposed with X‐rays (Figure 4c; Figure S13b, Supporting Information). Moreover, eighty percent of the mice survived within 60 days after the combined treatment of CCL injection and X‐rays exposure. In contrast, mice in control groups died within 30 days (Figure 4d). Meanwhile, hematoxylin‐eosin (H&E) staining was used to analyze the level of cell damage in the corresponding primary tumor sections collected from different groups. As predicted, CCL injection plus X‐rays exposure exhibited the most severe cell damage (Figure 4e). In addition, there was no significant change in body weight of all mice during the whole treatments (Figure S14a, Supporting Information). The major normal organs including heart, liver, spleen, lung, and kidney were collected from PBS‐treated and CCL plus X‐rays‐treated mice, and used for H&E staining analysis. There was no noticeable organ damage in the treated mice, preliminarily indicating the excellent biocompatibility and safety of our anti‐tumor strategy (Figure S14b, Supporting Information).

The inhibition of tumor growth depended not only on the enhanced radiotherapy, but was also attributed to the strengthened systemic antitumor immunotherapy, especially for the distant tumors.^[^ ^20^ ^]^ First of all, radiotherapy could cause ICD of primary tumors and these nanoparticles promoted ICD of tumors, which was confirmed in in vitro experiments. Therefore, we carried out the immunofluorescence staining of frozen sections of the primary tumor for CRT, HSP‐70, and HSP‐90, which are considered as specific signaling molecules for ICD. The fluorescence intensity was the highest in the CCL plus X‐rays‐treated tumor, suggesting that our antitumor strategy induced a significant ICD (Figure 4f; Figure S15, Supporting Information). ICD can promote the presentation of tumor specific antigens by DC and then trigger the potent systemic T‐cell‐mediated antitumor immunotherapy.

### 2.6 Remodeling the Immune Microenvironment of Tumors by CCL‐Mediated Radiotherapy

**Figure 5:**
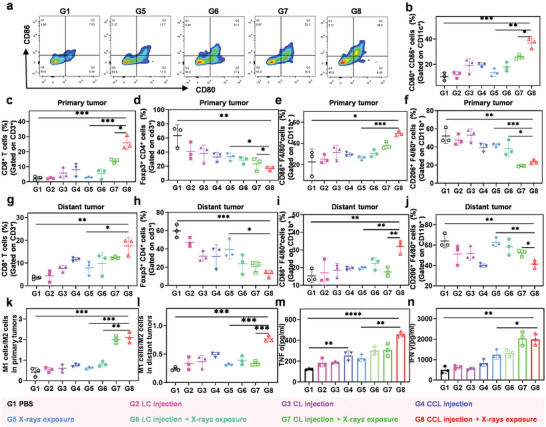
Remodeling tumor immune microenvironment by CCL plus X‐rays exposure. a,b) The representative flow cytometric plots (a) and the proportion of mature DCs (b) in the lymph nodes adjacent to primary tumors. c,d) The proportion of CTLs (CD3^+^CD8^+^ T cells) (c) and Treg cells (CD4^+^Foxp3^+^ T cells) (d) in primary tumors. e,f) The proportion of M1 macrophages (e) and M2 macrophages (f) in primary tumors. g,h) The proportion of CTLs (g) and Treg cells (h) in distant tumors. i,j) The proportion of M1 (i) and M2 macrophages (j) in distant tumors. k,l) The ratio of M1/M2 macrophages in primary tumors (k) and distant tumors (l). m,n) IFN‐γ (m) and TNF‐α (n) expression in the serum of mice in various groups. Data are presented as mean ± s.d. (n = 3). All data are analyzed by one‐way ANOVA. *, P<0.05, **, P < 0.01, ***, P < 0.001, ****, P < 0.0001.

Inspired by CCL‐changed content of H^+^ and Ca^2+^ and enhanced ICD, we next studied the anticancer immune response induced by our enhanced radiotherapy. In order to elucidate the mechanism, we prepared 8 groups of CT26 tumor‐bearing Balb/c mice to receive one round treatment as above, and the mice were sacrificed 3 days after the whole treatment. The primary tumors, their adjacent groin inguinal lymph nodes and the distant tumors were collected and analyzed by flow cytometer. First, we performed immunoassay for DC maturation. It was demonstrated that the PBS and plain nanoparticles had little effect on DC maturation in lymph nodes, while the frequency of DC maturation was significantly increased in the mice treated with CCL plus X‐rays exposure, owing to the enhanced ICD and Ca^2+^ content (**Figure** **5**a,b). In the T cell‐mediated anti‐tumor immune response, CTLs were the main effector cell and regulatory T cells (Tregs) were the main immunosuppressive cell. The mature DC cells would present the antigens to T cells and differentiate T cells into CTLs. Meanwhile, the enhanced pH of tumor microenvironment might decrease the tumor infiltration of various immunosuppressive cells .^[^ ^21^ ^]^ In our study, compared to those of individual nanoparticles‐treated mice, primary tumors treated by CCL plus X‐rays exposure showed a higher percentage of cytotoxic T cells (25.9±5.0%) and a lower abundance of Treg cells (17.0±3.1%) (Figure 5c,d; Figures S16a and S17a, Supporting Information). Similarly, the enhanced CTLs (17.5±4.2%) and decreased Tregs (13.0±4.3%) in the distant tumors were also found, suggesting the potent and systemic T cells‐mediated antitumor immune response in the mice treated with CCL plus X‐rays exposure (Figure 5g,h; Figures S16b and S17b, Supporting Information).

Besides, the macrophage polarization in the primary and distant tumors was also analyzed. The flow cytometry results revealed that CCL‐mediated radiotherapy could activate the innate anti‐tumor immunity via increasing the number of M1 macrophages and inhibiting the differentiation of immunosuppressive M2 macrophages in the primary tumors (Figure 5e,f; Figures S18a &S19a, Supporting Information). Similarly, the enhanced M1 and reduced M2 could be obtained in the distant tumors for the CCL plus X‐rays‐treated mice (Figure 5i,j; Figures S18b and S19b, Supporting Information). The ratio of M1 and M2 was calculated and was significantly enhanced by CCL‐mediated radiotherapy, much higher than in the other groups, indicating the strong macrophage‐mediated anti‐tumor immune response in mice treated with CCL and X‐rays exposure (Figure 5k,l). Notably, radiotherapy alone did not significantly induce the change of macrophage polarization in primary and distant tumors, while CCL‐mediated radiotherapy induced higher M1 and lower M2 than CCL injection alone. This might attribute to the radiotherapy‐induced calcium overload in the CCL‐treated mice, which could promote the macrophage polarization from M2 to M1. Furthermore, we found that CCL‐mediated radiotherapy also resulted in a significant increase in the secretion of cytotoxic IFN‐γ and TNF‐α by analyzing plasma component (Figure 5m,n). In summary, CCL‐mediated radiotherapy could induce a powerful anti‐tumor immune response by strengthening the T cells and macrophages‐mediated systemic anti‐tumor immunotherapy.

### 2.7 The Combined Effect of CCL‐Enhanced Radiotherapy and αPD‐L1

**Figure 6:**
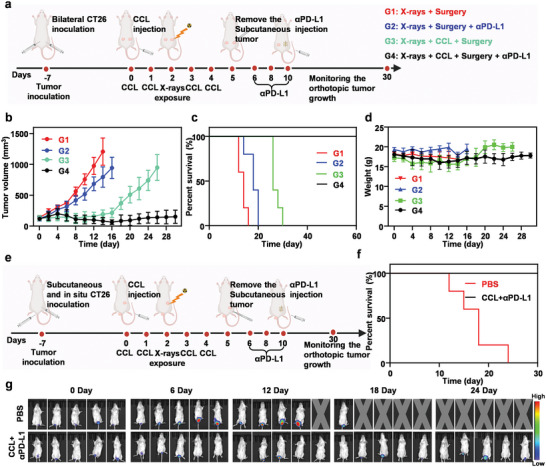
The combined effect of CCL‐enhanced radiotherapy and αPD‐L1. a) Schematic illustration of the experimental schedule indicating the combined effect of CCL‐enhanced radiotherapy and αPD‐L1 on distant tumors. The dose of the calcium, CAI and αPD‐L1 was 10, 2.5, and 1 mg kg^−1^ for each single injection. b) Tumor volume of distant tumors in mice in various groups. c) Survival curves of mice after various treatments. d) Body weight of mice in various groups. Data are presented as mean ± s.d. (n = 5). e) Schematic illustration of the experimental schedule indicating the combined effect of CCL‐enhanced radiotherapy and αPD‐L1 on orthotopic tumors. The dose of the calcium, CAI and αPD‐L1 was 10, 2.5, and 1 mg kg^−1^ for each single injection. f) Survival curves of mice after various treatments (n = 5). g) In vivo bioluminescent images to track the growth and metastases of CT26‐Luc cells after various treatments.

It is reported that radiotherapy could up‐regulate the expression of PD‐L1 in tumors, thus weakening the anti‐tumor effect of the T cells‐mediated immunotherapy.^[^ ^22^ ^]^ Therefore, frozen slices of distant tumors of mice with different treatments were stained with αPD‐L1 antibody. Immunofluorescence results showed that the expression of PD‐L1 in distant tumors collected from mice treated with CCL and X‐rays exposure was significantly up‐regulated (Figure S20a,b, Supporting Information). Inspired by excellent performance in anti‐tumor immunity, CCL‐enhanced neoadjuvant radiotherapy may have a better therapeutic effect for distant tumors when combined with αPD‐L1 therapy. When tumor volume reached ≈75 mm^3^, twenty Balb/c mice with bilateral tumor were randomly divided into 4 groups (n = 5) and received following treatments: (G1) X‐rays exposure+ surgery; (G2) X‐rays exposure + surgery + αPD‐L1; (G3) CCL injection + X‐rays exposure + surgery; (G4) CCL injection + X‐rays exposure + surgery + αPD‐L1 (**Figure** **6**a). The tumor growth curves showed that neoadjuvant radiotherapy alone or radiotherapy plus αPD‐L1 had a limited inhibitory influence on the distant tumors, while CCL‐enhanced radiotherapy could inhibit the growth of the distant tumor and the distant tumors were almost completely eliminated when CCL‐enhanced radiotherapy was combined with αPD‐L1 (Figure 6b). As a result, all of the tumor‐bearing mice treated with CCL‐enhanced radiotherapy and αPD‐L1 were survival in 60 days, showing the ideal therapeutic outcome by our strategy (Figure 6c). Moreover, there was no remarkable body weight loss for the treated mice, suggesting the safety of our anti‐tumor strategy (Figure 6d). In addition, we constructed an orthotropic rectal tumor model using firefly luciferase‐expressing CT26 cells, and investigated the therapeutic effect of our strategy on the distant orthotropic rectal tumors (Figure 6e). Bioluminescent imaging was used to monitor tumor growth in each group. CCL‐enhanced radiotherapy plus αPD‐L1 could significantly inhibit the growth and metastasis of rectal tumors and prolong the long‐term survival time of mice, in sharp contrast to the control group (Figure 6f&g). Simultaneously, there was no significant change in body weight during the whole treatment, indicating the biosafety of our combination therapy again (Figure S21, Supporting Information).

## 3 Conclusion

In summary, we successfully synthesized a pH‐responsive calcium carbonate nano‐drug (CaCO_3_/CAI@Liposome, CCL) to accurately regulate Ca^2+^ content and pH value in and out of tumor cells for enhanced tumor radio‐immunotherapy. CCL could release Ca^2+^ and CAI and deplete extracellular H^+^ in tumor microenvironment. Meanwhile, the released CAI from CCL prevented the exchange of H^+^ inside and outside cells by inhibiting carbonic anhydrase, resulting in the enhanced intracellular H^+^, which could further induce the calcium influx in tumor cells, and reversed tumor acidic microenvironment. The enhanced intracellular H^+^ and Ca^2+^ as well as reduced extracellular H^+^ improved the outcome of radiotherapy both in vitro and in vivo. Moreover, CCL together with radiotherapy could trigger potent systemic anti‐tumor immune response by amplifying the radiotherapy‐induced ICD and strengthening the T cells and macrophages‐involved antitumor immunotherapy. Finally, αPD‐L1 was used to block the up‐expressed PD‐L1 in the tumors treated with CCL‐enhanced radiotherapy. This combined therapy could effectively inhibit the growth of distant/orthotropic tumors, and prolong the survival of the tumor‐bearing mice in both of distant tumor model and in situ colon cancer model. Therefore, CCL possessed the great promise to enhance the therapeutic outcome of radiotherapy and immune checkpoint blocking therapy against multiple potential tumor types, providing an effective and safe radiotherapy to colorectal cancer.

## Conflict of Interest

The authors declare no conflict of interest.

## Supporting information

Supporting Information

## Data Availability

The data that support the findings of this study are available from the corresponding author upon reasonable request.
