# Supplementary material for: Regulation of Ion Homeostasis for Enhanced Tumor Radio‐Immunotherapy
Source: Adv Sci (Weinh). 2023 Sep 22;10(32):2304092. doi: 10.1002/advs.202304092 (PMC10646238; doi:10.1002/advs.202304092)
Supplement: Supplementary file 1 — Supporting Information [file ADVS-10-2304092-s001.pdf]

ADVANCED SCIENCE
Open Access

Supporting Information

for *Adv. Sci.*, DOI 10.1002/advs.202304092

Regulation of Ion Homeostasis for Enhanced Tumor Radio-Immunotherapy

Rui Qian, Xuan Yi, Teng Liu, Hua Chen, Yuhong Wang, Lin Hu, Lingchuan Guo, Kai Yang* and Haijun Deng**

Supplementary Materials

Regulation of Ion Homeostasis for Enhanced Tumor Radio-Immunotherapy

Rui Qian, Xuan Yi, Teng Liu, Hua Chen, Yuhong Wang, Lin Hu, Lingchuan Guo, Kai Yang*, Haijun Deng**

Prof. H. Deng, Mr. R. Qian,
Department of General Surgery and Guangdong Provincial Key Laboratory of Precision Medicine for Gastrointestinal Tumor, Nanfang Hospital, The First School of Clinical Medicine, Southern Medical University, Guangzhou, 510000, China.

Prof. K. Yang, Dr. T. Liu, Dr. L. Hu, Mr. H. Chen,
State Key Laboratory of Radiation Medicine and Protection, School of Radiation Medicine and Protection and School for Radiological and Interdisciplinary Sciences (RAD-X), Collaborative Innovation Center of Radiation Medicine of Jiangsu Higher Education Institutions, Soochow University, Suzhou, Jiangsu, 215123, China.

Prof. X. Yi,
School of Pharmacy, Jiangsu Key Laboratory of Inflammation and Molecular Drug Targets, Nantong University, Nantong, Jiangsu, 226001, China.

Prof. K. Yang, Prof. L. Guo, Miss. Y. Wang,
Department of Pathology, the First Affiliated Hospital of Soochow University, Suzhou, China

E-mail: xuanyi@ntu.edu.cn, kyang@suda.edu.cn, navyd999@163.com

Materials and methods

Materials. Acetazolamide was purchased from MedChemExpress. Calcium chloride anhydrous and ammonium bicarbonate were purchased from Aladdin. 1, 2-dioleoyl-sn-glycero-3-phosphate (sodium salt) (DOPA) was purchased from Macklin. Cholesterol was purchased from J&k Scientific. 1, 2-dihexadecanoyl-sn-glycero-3-phosphocholine (DPPC) was purchased from Xi'an ruixi Biological Technology Co. 1, 2-distearoyl-snglycero-3-phosphoethanolamine-N- (methoxy (polyethylene glycol)-5000) (DSPE-PEG_{5000}) was purchased from Laysan Biological Co., Ltd. Absolute ethyl alcohol was purchased from Yonghua Chemical Co., Ltd.

Synthesis of $CaCO_3$/CAI@Liposome nanoparticles. $CaCO_3$/CAI@Liposome (CCL) was synthesized via a gas diffusion reaction. Typically, 150 mg of calcium chloride anhydrous and 10 mg of acetazolamide were added to a beaker containing 100 ml of ethanol covered with tinfoil full of small holes. Next, 5 g of ammonium bicarbonate was added to another beaker. The two beakers were placed in an airtight container at 40 °C for 24 h, and then the solution was centrifuged at 8000 rpm for 20 min to collect $CaCO_3$/CAI (CC). The obtained CC was dispersed in ethanol and stored at 4 °C for future use.
In order to increase the biocompatibility of CC, 20 mg of CC dispersed in 5 ml anhydrous ethanol and 2 mg of DOPA dissolved in 1 ml of chloroform were mixed ultrasonically for 30 min in a water bath at 37 °C. The DOPA-modified CC was centrifugally purified by centrifugation. Next, the CC coated with DOPA was dispersed in a chloroform solution pre-containing 2 mg of cholesterol, 4 mg of DPPC, and 8 mg DSPE-PEG_{5000}, obtaining $CaCO_3$/CAI@Liposome (CCL). After stirring at room temperature for 12 h, chloroform was removed with a rotary evaporator and the remaining nanoparticles were hydrated with PBS under ultrasound. Finally, the synthesized CCL was collected, purified, and stored at 4 °C.

Characterization of $CaCO_3$/CAI@Liposome (CCL). The morphology of CC and CCL at different pH values was examined using transmission electron microscopy (TEM, JEM-2100 microscope). The hydrodynamic diameters of $CaCO_3$/CAI (CC), CAI-loaded liposome (CL), liposome-coated $CaCO_3$ (LC) and $CaCO_3$/CAI@Liposome (CCL) was measured by a Zetasizer Nano ZS90. The absorbance spectra of the nanoparticles were detected by a UV-Vis-NIR spectrometer (GENESYS 50).

CAI or Ca^{2+} release from CCL nanoparticles. In order to monitor the release efficiency of CAI or calcium ions from CCL at different pH values, 2 mg of CCL was added into dialysis bags (MWCO=3kDa) and placed into buffer solutions with pH

values of 7.4 and 5.8 at 37 °C. 1 ml dialysate was collected at the predetermined time. The content of calcium ions was measured by inductively coupled plasma mass spectrometry (ICAP7200) and the content of CAI was measured by UV-Vis-NIR spectrometer.

Cell experiments. CT26 mouse colon cancer cell line (CT26) and RAW 264.7 were obtained from Cell Source Center, Chinese Academy of Science (Shanghai, China). All cell lines were cultured under standard conditions. To verify the cytotoxicity of CCL, CT26 cells were inoculated into 96-well plates at a density of 1×10^4 per well, and then treated with different concentrations of LC, CL and CCL for 24 h. Finally, the relative cell viability was measured by multifunctional microplate reader (Synergy NEO) using Cell Counting Kit-8 (CCK8).

To evaluate the cell uptake, CT26 cells pre-inoculated into 6-well plates at a density of 2×10^5 per well, were cultured with DiD-labeled CCL (CAI: 100 μg/mL), and then collected at appointed time. A flow cytometer (BD FACSVerse, USA) was used to detect the fluorescence of DiD in CT26. To verify the influence of nanoparticles on the colony formation ability of cells, CT26 per-cultured with LC, CL or CCL for 24 h were seeded into 6-well plates at the density of 500 cells per well. After 14 days of cultivation, the colonies were fixed with 3.7% formaldehyde, made transparent by methanol, and stained with 0.1% crystal violet. The number of stained colonies was counted to determine the colony formation ability.

In order to verify the changes in intracellular Ca^{2+} content and pH value, CT26 cells (10×10^4 cells per well) were cultured with LC, CL or CCL for 24 h and then collected for BCECF-AM and Flou-4AM staining, respectively. A confocal microscopy (FV1200, Olympus) was used to observe the fluorescence intensity of BCECF-AM, which is related to the pH value, and Flou-4AM, which is related to intracellular calcium ion concentration.

To determine the radiosensitization of CCL in vitro, CT26 cells were pre-seeded in 6-well plates at a concentration of 10^5 per well and then co-cultured with LC, CL or CCL for 24 h followed by X-rays irradiation (6 Gy). γ-H2AX (Ser139), calreticulin (D3E6), DAPI and corresponding anti-rabbit AlexaFluor-488 were applied to stain CT26 cells. The fluorescence intensity of γ-H2AX verified the degree of DNA damage, while the fluorescence intensity of calreticulin represented the progression of ICD.

Tumor model. In our experiments, all female Balb/c mice (6−8 weeks) were purchased from Beijing Vital River Laboratory Animal Technology Co., Ltd. and used strictly under the protocols approved by Soochow University Laboratory Animal Center. CT26 cells (3×10^6) dispersed in 50 μl PBS were injected in the back of mice to establish the subcutaneous tumor model. The experiments were started when the tumor volume was about 75 mm^3, and the mice were considered dead when the tumor volume was greater than 1000 mm^3.

Fluorescent imaging. To test the in vivo biodistribution of nanoparticles, DiD-labeled LC, CL or CCL was intravenously injected into mice bearing CT26 tumor (n=3). The in vivo fluorescence imaging of mice was performed by an in vivo imaging System (Lumina III) at different time points. After injection of 24 h, mice were sacrificed, and collected the major tissues and tumors for ex vivo bio-distribution analysis.

Tumor microenvironment regulation by CCL. To explore the effect of various nanoparticles on the pH value of tumor microenvironment, twelve mice with bilateral tumors were randomly divided into four groups (n=3). One side of the tumor was excised and broken down into a cellular homogenate with scissors, and its pH value was measured with a pH meter. Meanwhile, these mice were also intravenously injected with PBS, LC, CL or CCL, and the pH value of the contralateral tumor was measured by the same method described above at 24 h post injection. The concentration of intracellular H^+ and Ca^{2+} was tested in vitro using the same method on the frozen tumor slices collected from the tumor-bearing mice with the indicated treatments.

Tumor growth inhibition. To check the inhibition of primary and distant tumors by the indicated treatments, the bilateral CT26 tumor-bearing BALB/c mice were randomly allocated into eight groups (n = 5) and implemented with treatment protocol as follow when the tumor volume reached ~75 mm^3: 1) PBS; 2) X-rays exposure (6 Gy); 3) LC; 4) LC + X-rays exposure(6 Gy); 5) CL; 6) CL + X-rays exposure (6 Gy); 7) CCL; 8) CCL+ X-rays exposure (6 Gy). The dose of the calcium

and CAI was 10 mg/kg and 2.5 mg/kg, respectively. These treatments were carried out two rounds. The tumor volume was calculated using $a \times b^2/2$, and the body weight of the mice was recorded by balance every two days.

Immune cell proportion analysis. Mice with bilateral CT26 tumors were divided into eight groups (n = 3) and treated for one round as described above. 4 days later, the mice were sacrificed to collect the bilateral tumors and juxta-tumor lymph nodes. To verify the maturation of DC cells, single-cell suspensions of lymph nodes were stained with anti-CD11c-FITC (eBioscience, Clone: N418, 11-0114-82), anti-CD86-APC (eBioscience, Clone: GL1, 17-0862-82) and anti-CD80-PE (eBioscience, Clone: 16-10A1, 12-0801-82) antibodies. To study T cells infiltration, single-cell suspensions of tumors were stained with anti-CD3-FITC (eBioscience, Clone: 17A2, 11-0032-82), antiCD4-APC (eBioscience, Clone: GK1.5, 17-0041-83) and anti-CD8a-PE (eBioscience, Clone: 53-6.7, 12-0081-83) antibodies. To study Treg cells differentiation, single-cell suspensions of tumors were stained with anti-CD3-FITC (eBioscience, Clone: 17A2, 11-0032-82), antiCD4-APC (eBioscience, Clone: GK1.5, 17-0041-83) and anti-FOXP3-PE (eBioscience, Clone: FJK-16s, 12-5773-82) antibodies. All of these cells were analyzed by a flow cytometer (BD FACSVerse, USA).
For ELISA analysis, serum samples collected from the corresponding mice were diluted and analyzed. Tumor necrosis factor -alpha (TNF-α, Sino Biological) and γ interferon (IFN-γ, BD Biosciences) in serum were quantified by sandwich Elisa kit according to the manufacturer's instructions.

The combined therapy of CCL-enhanced radiotherapy and αPD-L1. To evaluate the combined efficacy of CCL-enhanced radiotherapy with αPD-L1, the bilateral CT26 tumor-bearing BALB/c mice were randomly allocated into 4 groups (n = 5) and received treatment as follow: 1) X-rays exposure + surgery; 2) X-rays exposure + surgery + αPD-L1; 3) CCL injection + X-rays exposure + surgery; 4) CCL injection + X-rays exposure + surgery + αPD-L1. The dose of the calcium and CAI was 10 mg/kg and 2.5 mg/kg, respectively, and the dose of X-rays was 6 Gy. The volume of distant tumors and the weight of mice were measured every two days.
To analyze orthotropic colon carcinoma inhibition, we first constructed an orthotropic tumor model by injecting 3×10^6 Luciferase-CT26 cells in the mucosa of the colon over the dentate line directly. Then, 10 mice with subcutaneous and orthotropic CT26 tumors were divided into two groups (n = 5) and treated as follow: 1) X-rays exposure+ surgery; 2) CCL injection + X-rays exposure + surgery + αPD-L1. Tumors were monitored by an in vivo imaging System (Lumina III) every 6 days and the weight of mice were measured every two days.

Statistical Analysis. The experimental results were expressed as mean values ± SD. Sample size (n) for each statistical analysis was provided in the figure caption. A two-tailed Student's t-test or a one-way ANOVA was used for the analysis of statistical difference between two groups in GraphPad Prism9. $P<0.05$ was considered statistically significant. ns, $P>0.05$; *, $P<0.05$; **, $P < 0.01$; ***, $P < 0.001$; ****, $P < 0.0001$.

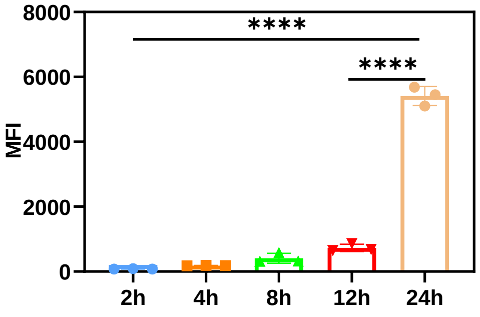

Figure S1. MFI (mean fluorescence intensity) analysis of time-dependent cellular uptake of DiD-labeled CCL in CT26 cells. Data are presented as mean ± s.d. (n = 3). All data are analyzed by one-way ANOVA. ****, P < 0.0001.

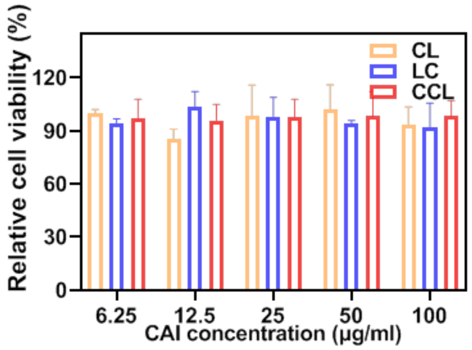

Figure S2. The viability of NIH-3T3 cells incubated with different concentrations of CL, LC or CCL. Data are presented as mean ± s.d. (n = 3).

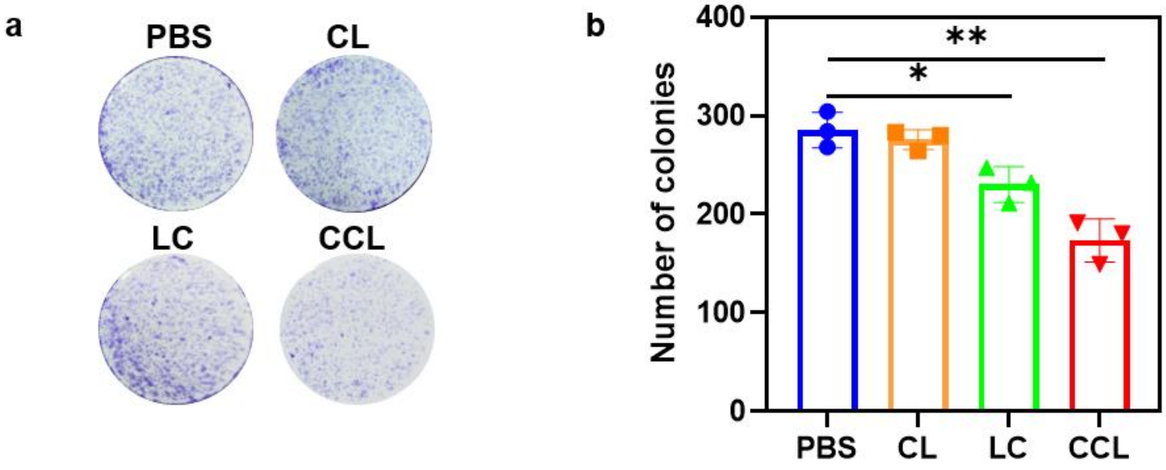

Figure S3. (a) Colony formation of CT26 cells incubated with PBS, CL, LC or CCL. (b) Number of CT26 colonies with indicated treatment. Data are presented as mean ± s.d. (n = 3). All data are analyzed by one-way ANOVA. *, P<0.05, **, P < 0.01, ***, P < 0.001, ****, P < 0.0001.

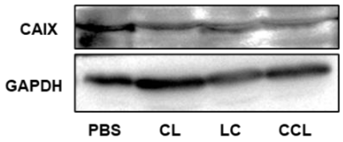

Figure S4. The representative western blotting image showing the expression of CAIX in CT26 cells with indicated treatments.

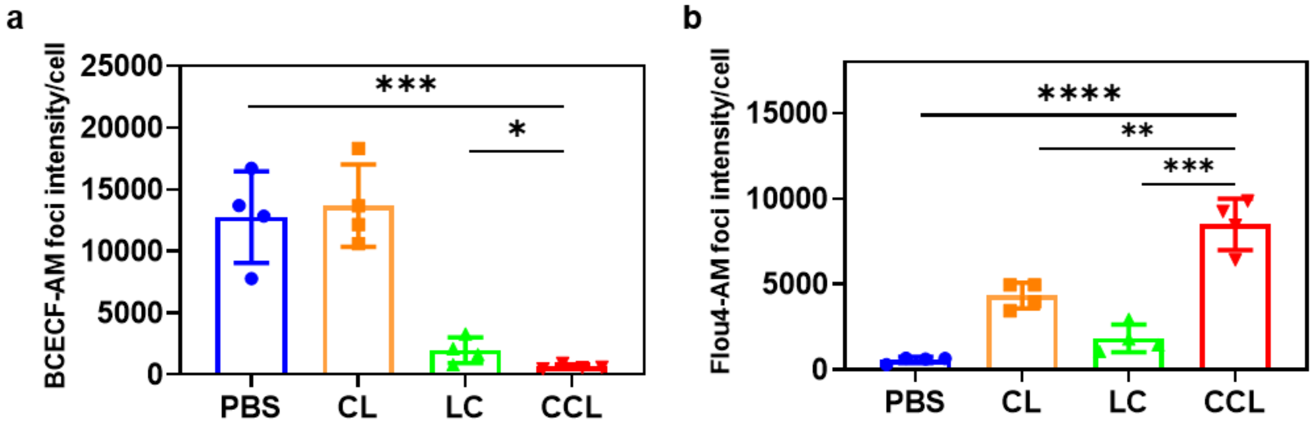

Figure S5. (a, b) Corresponding semiquantitative analysis of BCECF-AM and Flou4-AM fluorescence intensity of CT26 cells incubated with PBS, CL, LC or CCL. Data are presented as mean ± s.d. (n = 4). All data are analyzed by one-way ANOVA. *, P<0.05, **, P < 0.01, ***, P < 0.001, ****, P < 0.0001.

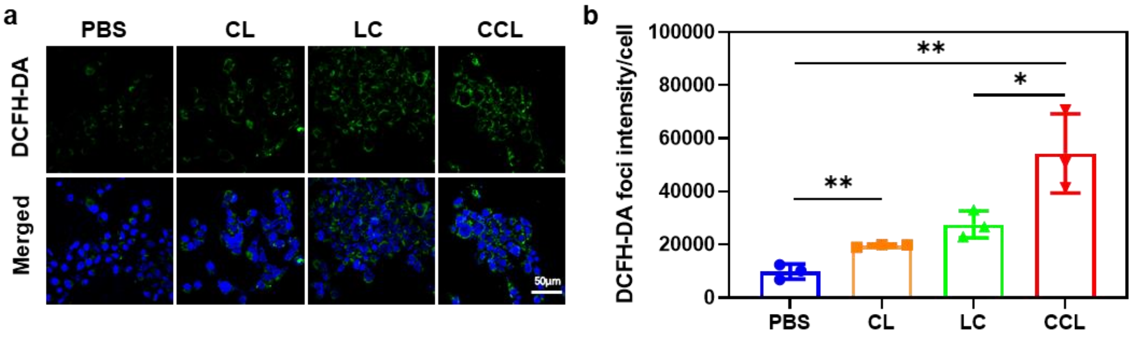

Figure S6. (a) DCFH-DA fluorescent images of CT26 cells incubated with PBS, CL, LC or CCL. The scale bar is 50 µm. (b) Corresponding semi-quantitative analysis of DCFH-DA fluorescence intensity of CT26 cells incubated with PBS, CL, LC or CCL. Data are presented as mean ± s.d. (n = 3). All data are analyzed by one-way ANOVA. *, P<0.05, **, P < 0.01.

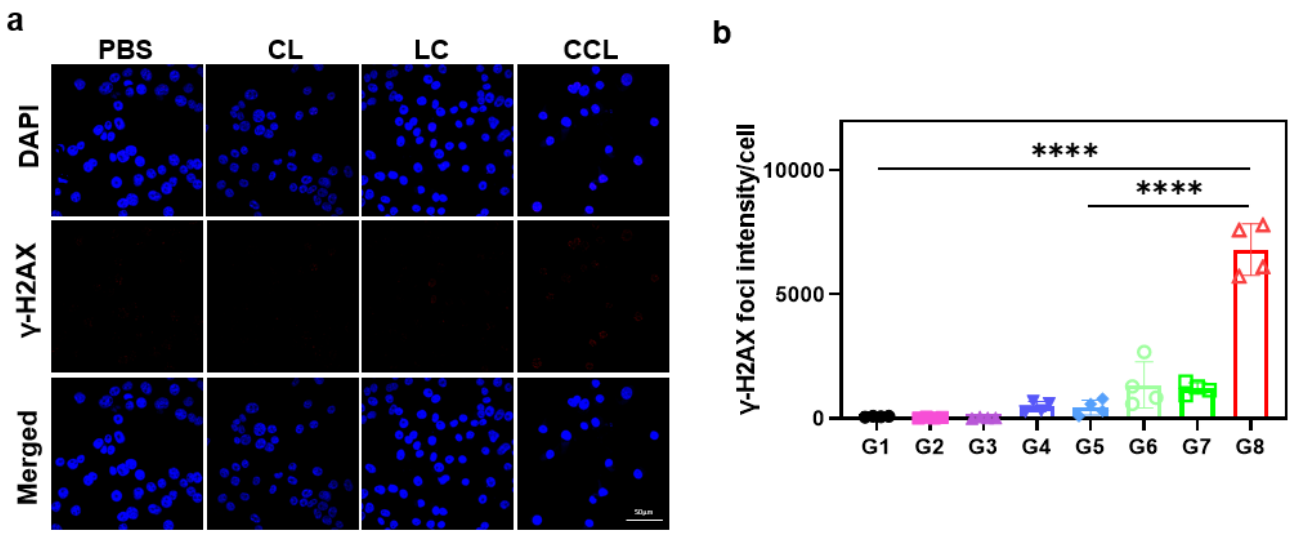

Figure S7. (a) γ-H2AX fluorescence images of CT26 cells incubated with PBS, CL, LC or CCL under X-rays exposure. The scale bar is 50 μm. (b) Corresponding semiquantitative analysis of γ-H2AX fluorescence intensity of CT26 cells incubated with indicated treatment. Data are presented as mean ± s.d. (n = 4). All data are analyzed by one-way ANOVA. (a) Colony formation of CT26 cells incubated with PBS, CL, LC or CCL. (b) Number of CT26 colonies with indicated treatment. Data are presented as mean ± s.d. (n = 3). All data are analyzed by one-way ANOVA. *, P<0.05, **, P < 0.01, ***, P < 0.001, ****, P < 0.0001.

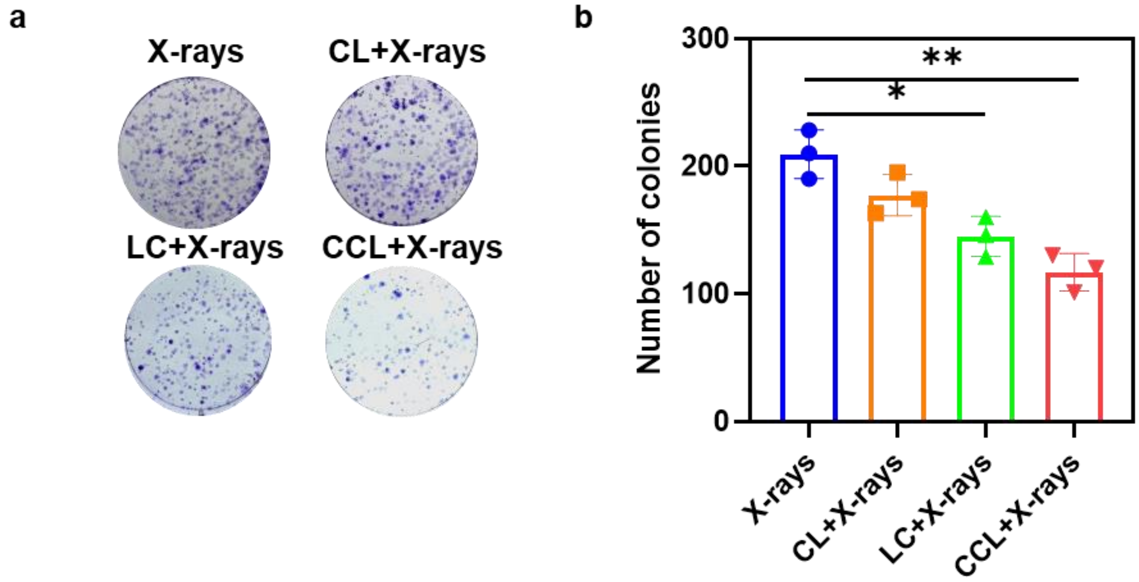

Figure S8. (a) Colony formation of CT26 cells incubated with PBS, CL, LC or CCL under X-rays exposure. (b) Number of CT26 colonies with indicated treatment. Data are presented as mean ± s.d. (n = 3). All data are analyzed by one-way ANOVA. (a) Colony formation of CT26 cells incubated with PBS, CL, LC or CCL. (b) Number of CT26 colonies with indicated treatment. Data are presented as mean ± s.d. (n = 3). All data are analyzed by one-way ANOVA. *, P<0.05, **, P < 0.01, ***, P < 0.001, ****, P < 0.0001.

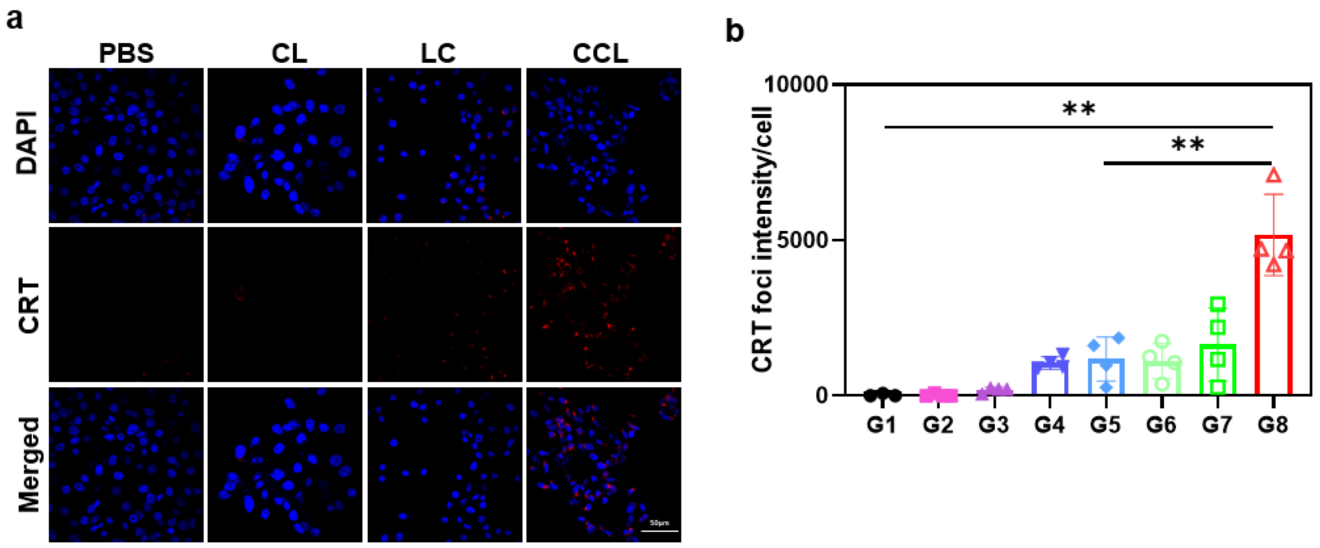

Figure S9. (a) CRT fluorescent images of CT26 cells incubated with PBS, CL, LC or CCL under X-rays exposure. The scale bar is 50 μm. (b) Corresponding semiquantitative analysis of CRT fluorescence intensity of CT26 cells incubated with indicated treatment. Data are presented as mean ± s.d. (n = 4). All data are analyzed by one-way ANOVA. *, P<0.05, **, P < 0.01, ***, P < 0.001, ****, P < 0.0001.

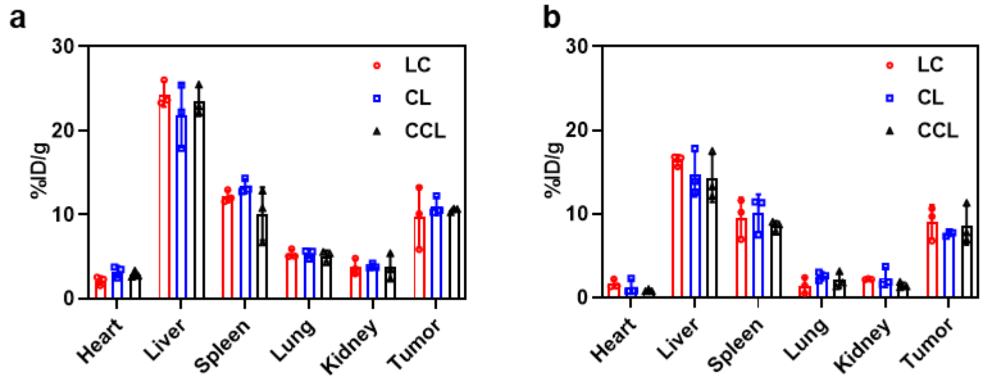

Figure S10. (a) Biodistribution profiles of CL, LC, CCL at 2 h post injection by recording the DiD fluorescence intensity of these homogenized organs and tumors (n = 3). (b) Biodistribution profiles of CL, LC, CCL at 24 h post injection by recording the DiD fluorescence intensity of these homogenized organs and tumors. Data are presented as mean ± s.d. (n = 3).

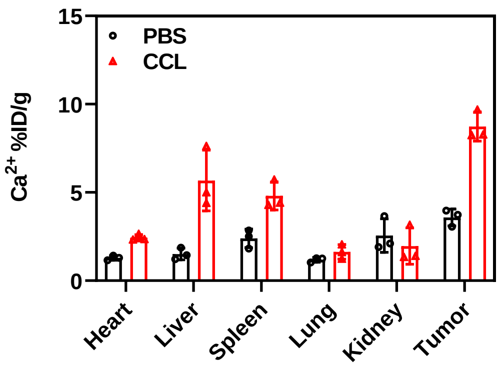

Figure S11. Biodistribution profiles of Ca^{2+} content at 24 h post intravenous injection of PBS or CCL. Data are presented as mean ± s.d. (n = 3).

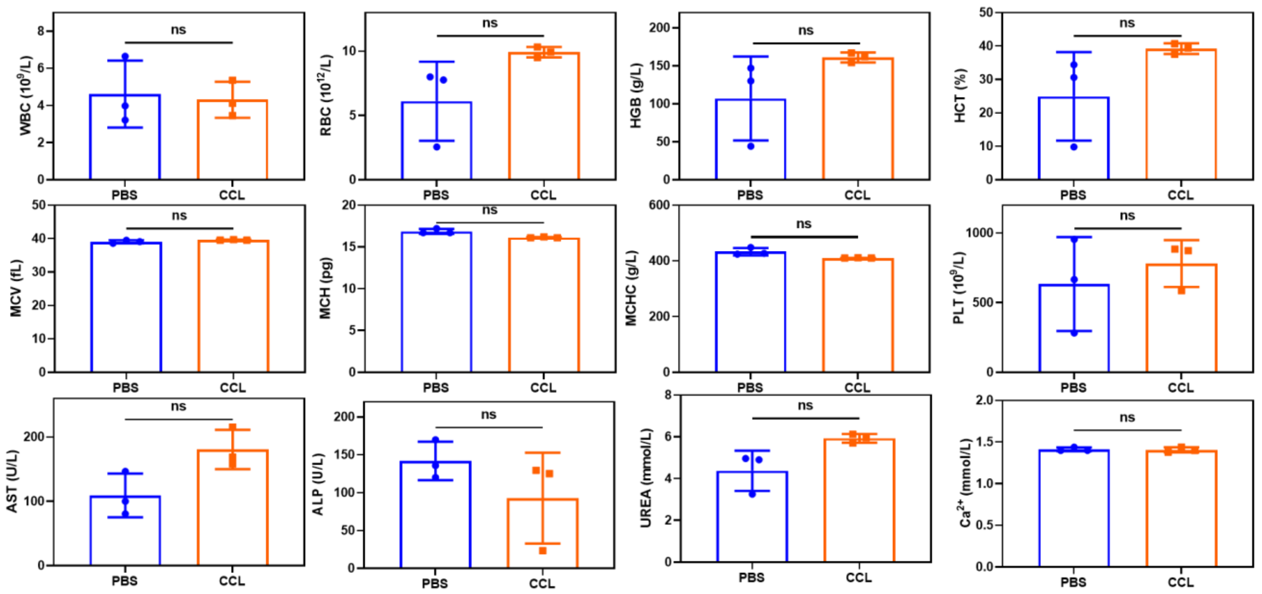

Figure S12. The in vivo biological security of CCL. Blood routine and blood biochemistry indicators including white blood cells (WBC), red blood cells (RBC), hemoglobin (HGB), hematocrit (HCT), mean corpuscular volume (MCV), mean corpuscular hemoglobin (MCH), mean corpuscular hemoglobin concentration (MCHC), and platelets (PLT), aspartate aminotransferase (AST), alkaline phosphatase (ALP), UERA levels, and blood Ca^{2+} concentration, were measured in the blood samples collected from healthy female Balb/c mice treated with PBS or CCL at day 3 post-injection. Data are presented as mean ± s.d. (n = 3). All data are analyzed by one-way ANOVA. ns, P>0.05.

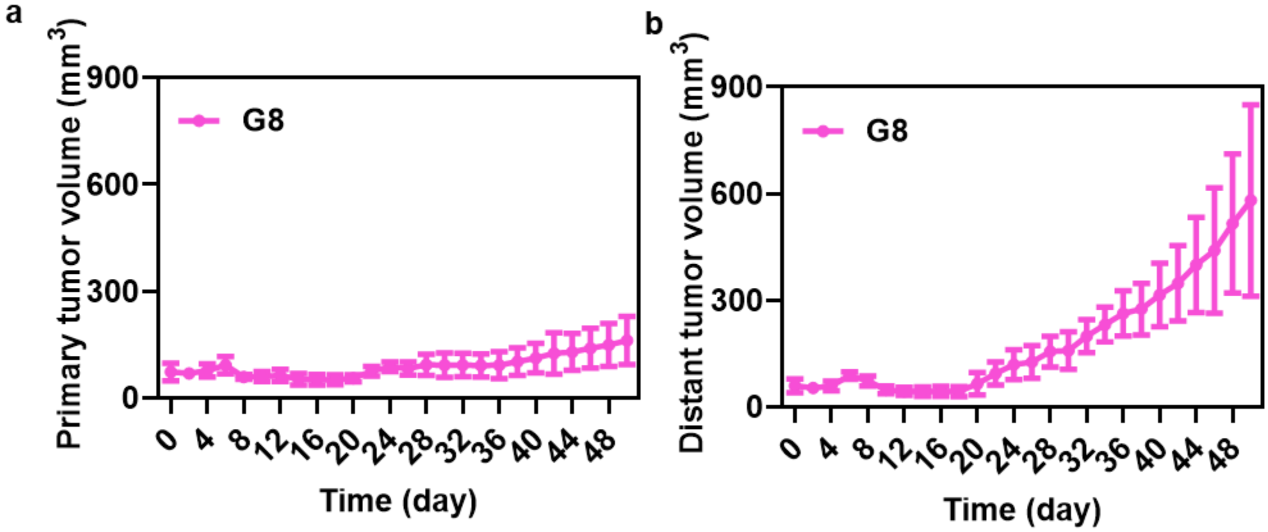

Figure S13. Tumor volume of primary and distant tumors on the mice in G8 group. Data are presented as mean ± s.d. (n = 5).

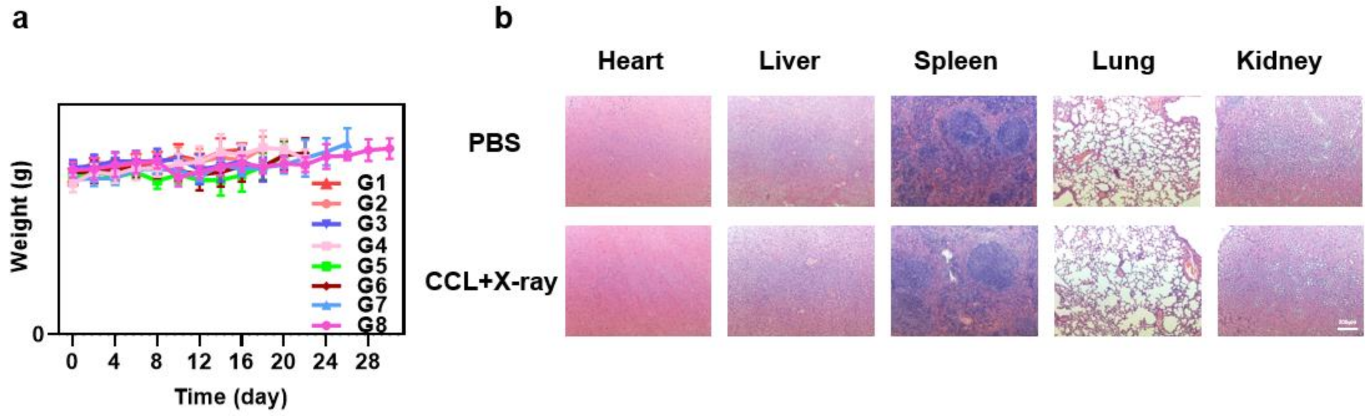

Figure S14. (a) Body weight curves of mice in various groups. Data are presented as mean ± s.d. (n = 5). (b) H&E-stained major organs in mice with indicated treatment. The scale bar is 200 μm.

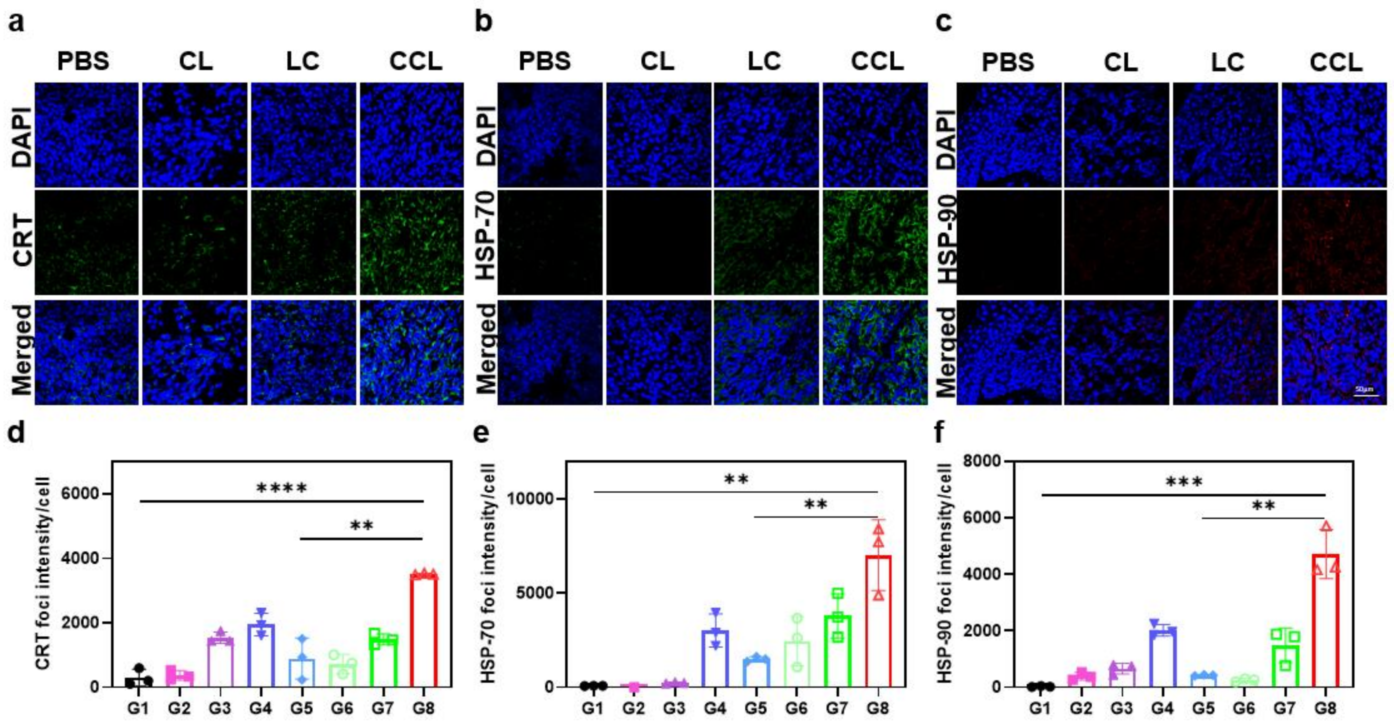

Figure S15. (a-c) Immunofluorescent slices of the CRT (a), HSP70 (b) and HSP90 (c) expression in primary tumors collected from mice treated with CL, LC or CCL. The scale bar was 50 μm. (d-f) Corresponding semiquantitative analysis of CRT (d), HSP70 (e) and HSP90 (f) fluorescence intensity of immunofluorescent slices in primary tumors. Data are presented as mean ± s.d. (n = 3). All data are analyzed by one-way ANOVA. *, P<0.05, **, P < 0.01, ***, P < 0.001, ****, P < 0.0001.

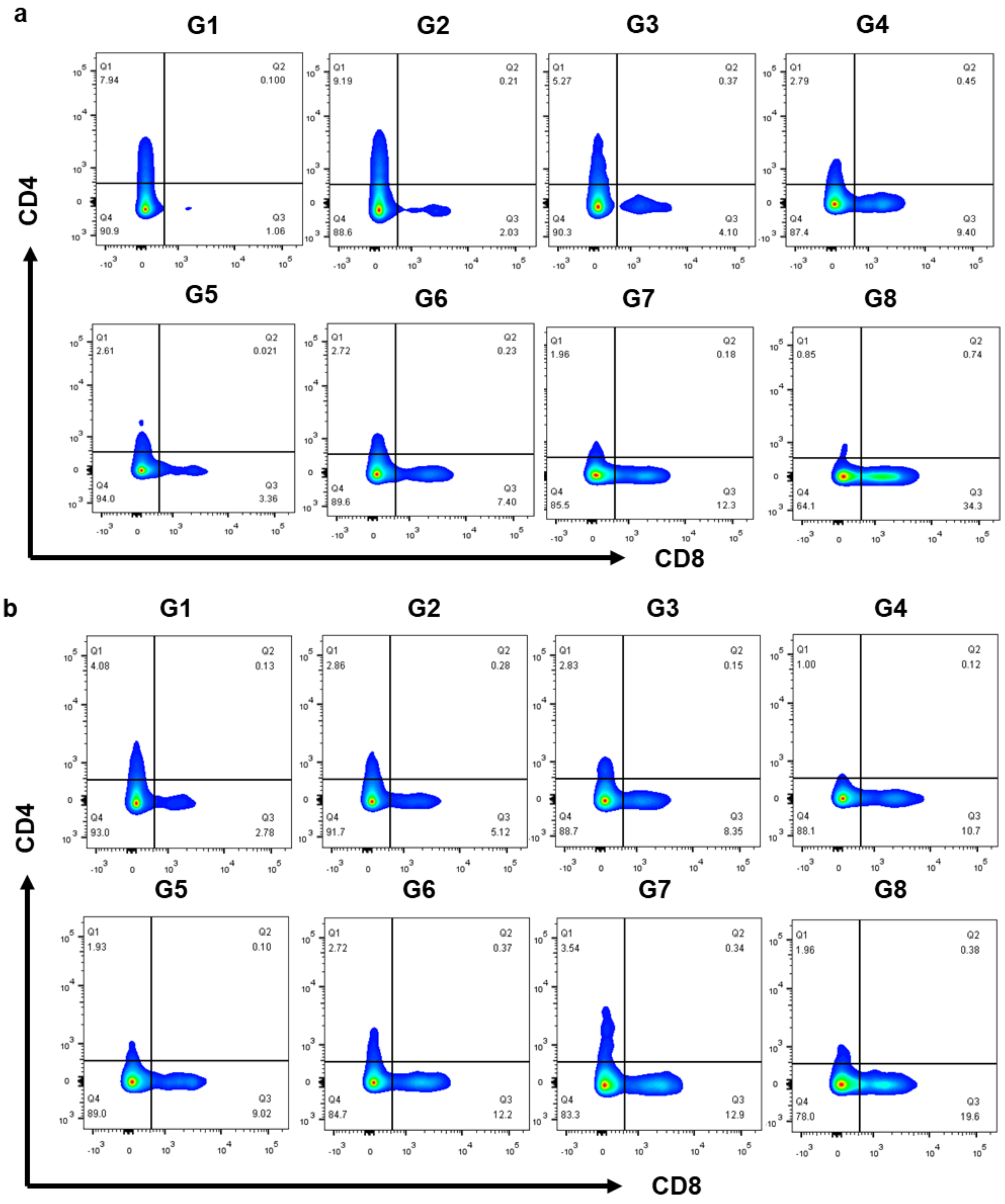

Figure S16. (a-b) Representative flow cytometric plots of CTLs ($CD3^+CD8^+$ T cells) in primary tumors (a) and distant tumors (b). (G1) PBS; (G2) LC injection; (G3) CL injection; (G4) CCL injection; (G5) X-rays exposure; (G6) LC injection + X-rays exposure; (G7) CL injection + X-rays exposure; (G8) CCL injection + X-rays exposure.

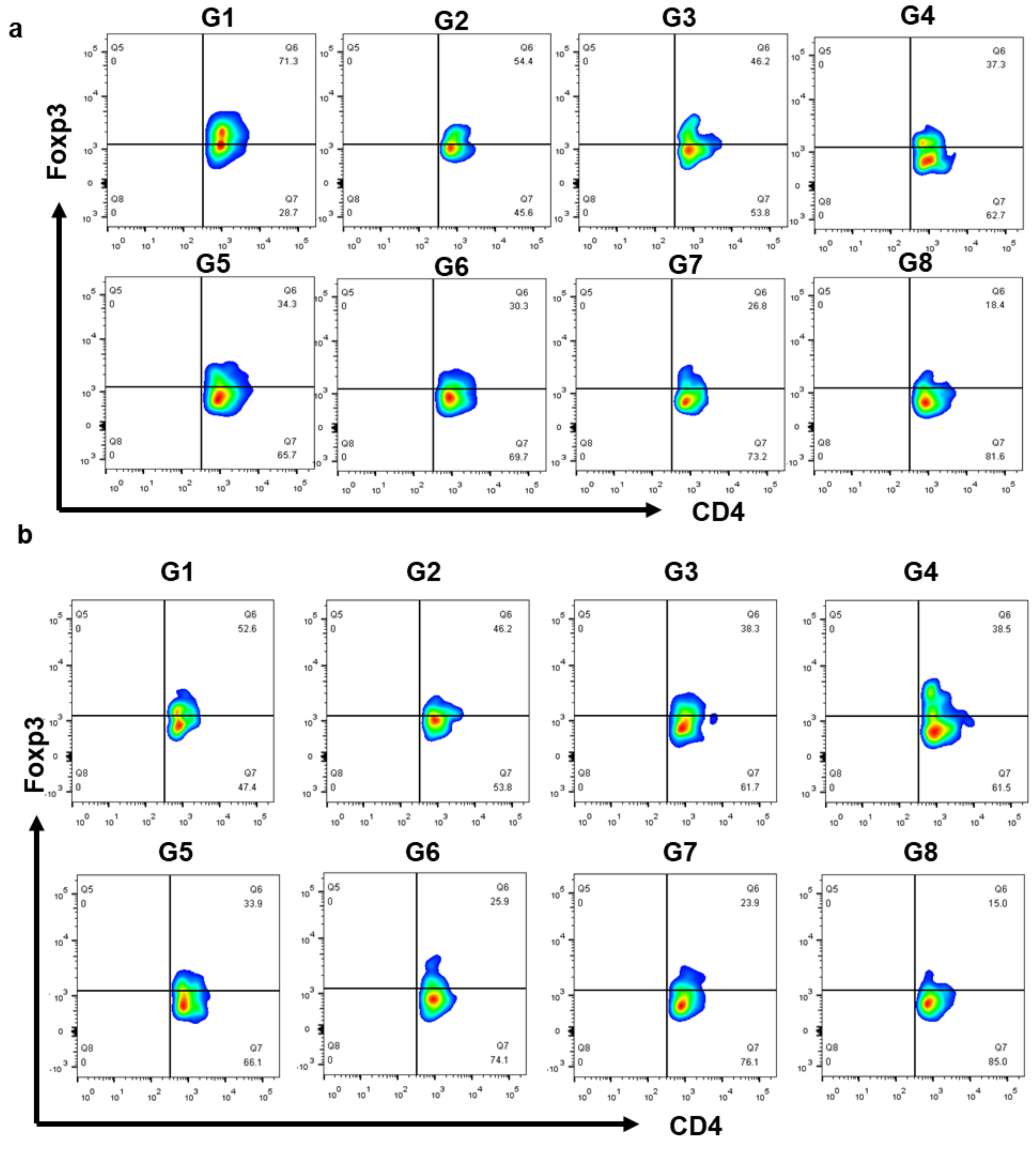

Figure S17. (a-b) Representative flow cytometric plots of Treg cells ($CD4^+Foxp3^+$ T cells) in primary tumors (a) and distant tumors (b). (G1) PBS; (G2) LC injection; (G3) CL injection; (G4) CCL injection; (G5) X-rays exposure; (G6) LC injection + X-rays exposure; (G7) CL injection + X-rays exposure; (G8) CCL injection + X-rays exposure.

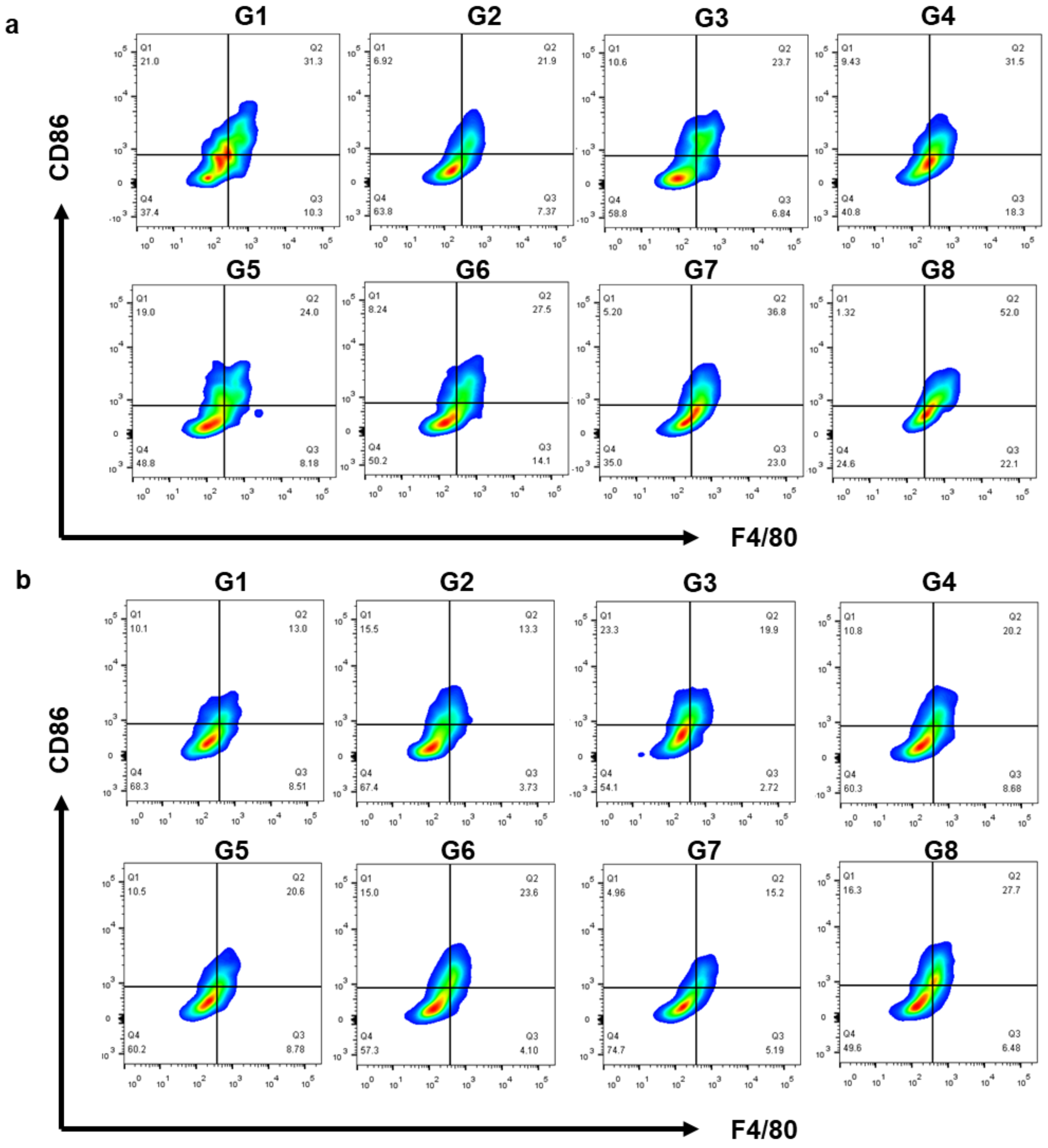

Figure S18. (a-b) Representative flow cytometric plots of M1 macrophages in primary tumors (a) and distant tumors (b). (G1) PBS; (G2) LC injection; (G3) CL injection; (G4) CCL injection; (G5) X-rays exposure; (G6) LC injection + X-rays exposure; (G7) CL injection + X-rays exposure; (G8) CCL injection + X-rays exposure.

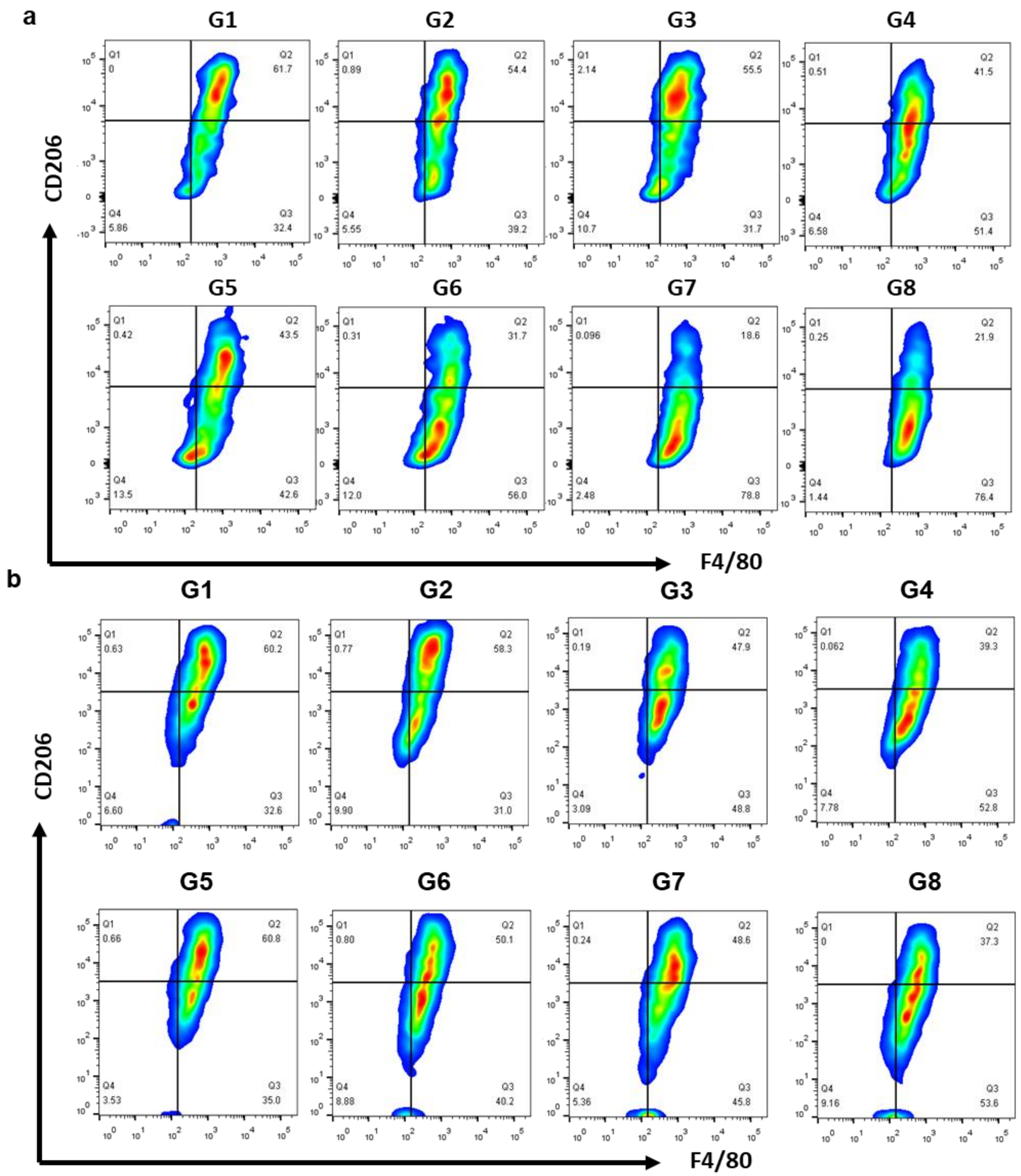

Figure S19. (a-b) The representative flow cytometric plots of M2 macrophages in primary tumors (a) and distant tumors (b). (G1) PBS; (G2) LC injection; (G3) CL injection; (G4) CCL injection; (G5) X-rays exposure; (G6) LC injection + X-rays exposure; (G7) CL injection + X-rays exposure; (G8) CCL injection + X-rays exposure.

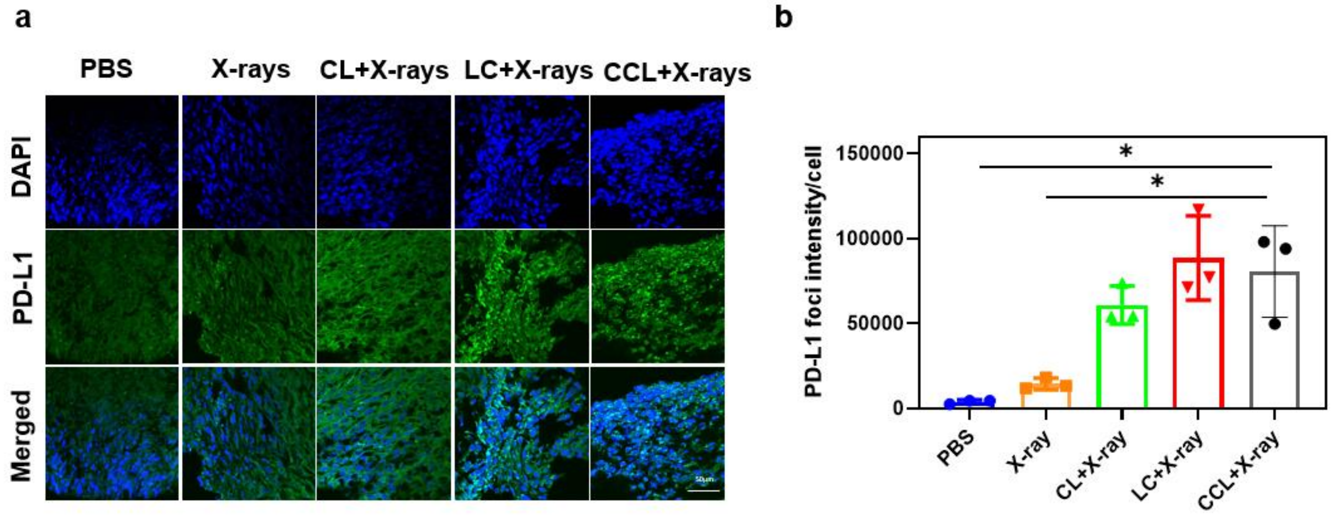

Figure S20. (a) Immunofluorescent slices of the PD-L1 expression in distant tumors collected from mice treated with CL, LC or CCL under X-rays exposure. (b) Corresponding semiquantitative analysis of PD-L1 fluorescence intensity of immunofluorescent slices in distant tumors. Data are presented as mean ± s.d. (n = 3). All data were analyzed by one-way ANOVA. Data are presented as mean ± s.d. (n = 3). *, P<0.05, **, P < 0.01, ***, P < 0.001, ****, P < 0.0001.

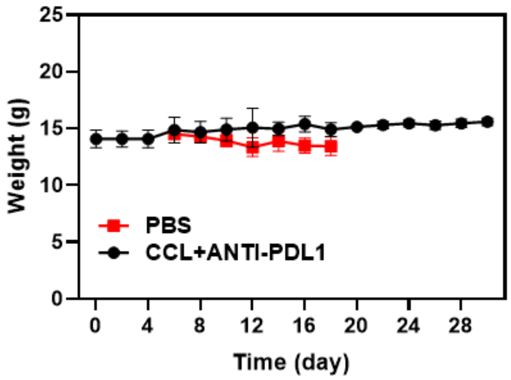

Figure S21. Body weight curves of mice in various groups. Data are presented as mean ± s.d. (n = 5).
